# Arthproliferins A–D, Four New Sesterterpenes from the Mangrove-Sediment-Derived Fungus *Arthrinium* sp. SCSIO41221

**DOI:** 10.3390/molecules28217246

**Published:** 2023-10-24

**Authors:** Bin Yang, Cuitian Li, Ying Chen, Yanchun He, Jianglian She, Xuefeng Zhou, Huangming Tao, Bo Peng

**Affiliations:** 1 CAS Key Laboratory of Tropical Marine Bio-Resources and Ecology, Guangdong Key Laboratory of Marine Materia Medica, South China Sea Institute of Oceanology, Chinese Academy of Sciences, Guangzhou 510301, China; 15071771508@163.com (Y.C.); heyanchun22@mails.ucas.ac.cn (Y.H.); sjlsjl0210@163.com (J.S.); xfzhou@scsio.ac.cn (X.Z.); 2 Marine Environmental Engineering Center, South China Sea Institute of Oceanology, Chinese Academy of Sciences, Guangzhou 510515, China; li_cuitian@163.com; 3 School of Traditional Chinese Medicine, Southern Medical University, Guangzhou 510515, China; taohm@smu.edu.cn; 4 Institute for Environmental and Climate Research, Jinan University, Guangzhou 511443, China

**Keywords:** *Arthrinium* sp., mangrove-sediment-derived fungus, sesterterpenes, cytotoxicity

## Abstract

Four new sesterterpenes, arthproliferins A–D (**1**–**4**), together with four known derivatives, were isolated and characterized from the mangrove-sediment-derived fungus *Arthrinium* sp. SCSIO41221. Their structures were determined using detailed nuclear magnetic resonance (NMR) and mass spectroscopic (MS) analyses. Some of the isolated compounds were evaluated for their cytotoxicity in vitro. The results revealed that terpestacin (**6**) exhibited significant activity with an IC_50_ value of 20.3 μM, and compounds **2** and **5** were found to show weak inhibitory effects against U87MG-derived GSCs.

## 1. Introduction

The mangrove forests are a complex ecosystem, and they are characterized by periodic tides that result in highly variable salinity and nutrient availability [1]. To adapt to the frequent and extreme environmental changes, an active microbial community, which constitutes the second-largest ecological group of the marine microbes, was formed under these special ecological conditions. Among the mangrove microbial community, mangrove-sediment-derived microbes not only play an essential role in creating and maintaining this biosphere, but also represent a rich source of secondary metabolites with novel structures and significant pharmacological activities, thus attracting a great deal of interest from scientists [2]. Several important reviews have shown that mangrove-associated fungi are the dominant producers of new natural products with pronounced biological activities [2,3]. Among these natural products of marine origin, the isolation of terpenoids has been exhaustively reported [4,5]. As a special subclass of terpenoids, marine-derived sesterterpenes with diverse skeletal types are of great attention due to their significant biological activities, such as antimicrobial, cytotoxic, anti-inflammatory, and protein tyrosine phosphatase B inhibitory activities [6,7,8].

Terpestacin, a bicyclic sesterterpene containing a transfused [3.0.13] bicyclic skeleton that includes a 15-membered macrocycle with three geometrically defined trisubstituted olefins, was first reported from the filamentous fungus *Arthrinium* sp. FA1744 (ATCC74132) in 1993 [9,10,11,12]. Heretofore, no more than 30 similar sesterterpenes have been obtained in nature. Examples include fusaproliferin from *Fusarium proliferatum* [13], fusaprolifins A and B from *Fusarium proliferatum* MA-84 [14], and 11-Epiterpestacin from *Bipolaris sorokiniana* NSDR-011 [15]. Although very few have been found, they have shown significant biological activities [16]. Great interest in these sesterterpenes has come from the finding that terpestacin was shown to effectively inhibit the formation of syncytia, the angiogenesis, without affecting the endothelial cell viability and the extracellular signal-regulated kinase activity [17,18]. Several total syntheses of terpestacin have been reported to date, and a series of active derivatives have also been designed and synthesized [17,19,20]. These results demonstrate that terpestacin analogues for treatment as anticancer and anti-HIV agents might offer promising research outcomes, and they encourage us that more effort is worth spending in this prospective field.

In our previous studies, our group has reported a range of new bioactive secondary metabolites from fungi associated with mangrove plants and soils, including bisabolanoic acid A [21], 1-methoxypestabacillin B [22], isochromophilones A−F [23], vaccinols A–S [24,25], 8-chlorine-5-hydroxy-2,3-dimethyl-7-methoxychromone, and 3,4-dichloro-1H-pyrrole-2,5-dione [26]. Some of them exerted various bioactivities, including cytotoxicity, anti-PTP1B, antienterovirus 71 (EV71), antituberculosis, anti-inflammatory, and antibacterial properties. During the course of our ongoing search for novel and bioactive compounds from filamentous fungi, the fungal strain *Arthrinium* sp. SCSIO41221, which has been isolated from a mangrove sediment sample from the South China Sea, was investigated. In the study presented here, the chemical investigation of an EtOAc extract of a solid culture led to the isolation of four new sesterterpenes—arthproliferins A–D (**1**–**4**)—and four known sesterterpenes—21-hydroxyterpestacin (**5**), terpestacin (**6**), fusaproliferin (**7**), and saponaroxin A (**8)**. We present herein the fermentation, isolation, structure elucidation, and activities of compounds **1**–**8.**

## 2. Results

**Figure 1:**
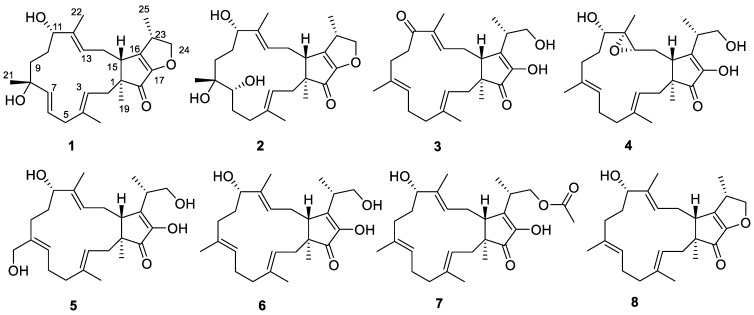
Structures of compounds **1**–**8.**

The rice solid culture of *Arthrinium* sp. SCSIO41221 was extracted using an EtOAc extract. Several chromatographic methods, including an MPLC with silica and an ODS gel column, as well as a semipreparative HPLC with a C-18 column, were used for the isolation of the eight terpestacin analogous sesterterpenes (Figure 1).

**Figure 2:**
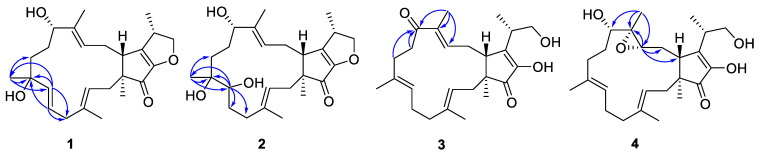
HMBC (arrows) correlations of **1**−**4**.

Compound **1** was isolated as a pale yellow amorphous solid. Based on the HRESIMS ion peak determined at an *m*/*z* of 423.2516 [M + Na]^+^ (calcd for 423.2506), the molecular formula was established as C_25_H_36_O_4_ and indicated eight degrees of unsaturation. The NMR data (Table 1 and Table 2) of **1**, with the aid of DEPT and HSQC (Figure S4) experiments, revealed resonances that were suggestive of a disubstituted double bond (*δ*_H_ 5.69 for H-6; *δ*_H_ 5.82 for H-7; *δ*_C_ 137.4 for C-6; and *δ*_C_ 126.2 for C-7), two trisubstituted double bonds (*δ*_H_ 5.26 for H-3; *δ*_H_ 5.68 for H-13; *δ*_C_ 120.8 for C-3; 137.4 for C-4; 136.5 for C-12; and 121.5 for C-13), a tetrasubstituted double bond (*δ*_C_ 150.8 for C-16 and 147.6 for the oxygenated C-17). Other characteristic signals included five methyl groups (*δ*_C_ values of 15.9, 27.0, 13.6, 16.9, and 13.4), six methylenes (including an oxymethylene), three methines (including an oxymethine), three quaternary carbons (including a ketone carbon and an oxygenated quaternary carbon). All of these ^1^H and ^13^C NMR resonances accounted for five degrees of unsaturation. The remaining three degrees of unsaturation indicated that **1** is a tricyclic compound. These data showed great similarities to those of saponaroxin A [27], except for an oxygenated quaternary carbon and a disubstituted double bond instead of a trisubstituted double bond and a methylene in the 15-membered ring. This assumption was supported by the correlation of Me-21 to C-7, C-8, and C-9, of H-7 to C-5, C-8, and Me-21, and of H-6 to C-5 and C-8 in the HMBC spectrum (Figure 2). Thus, the gross structure of **1** was established.

Compound **2** was isolated as a pale yellow oil. Based on the HRESIMS ion peak at an *m*/*z* of 441.2623 [M + Na]^+^ (calcd for 441.2611), the molecular formula was established as C_25_H_38_O_5_ and indicated seven degrees of unsaturation. Both the ^1^H and ^13^C NMR spectra of **2** showed a close similarity to those of **1** (Table 1 and Table 2). However, a close comparison of the ^13^C NMR spectroscopic data of **2** and **1** revealed some differences: one bisubstituted double bond in **1** was changed to an oxymethine and a methylene in **2**. This assumption was supported by the correlation of Me-21 to C-7, C-8, and C-9, as well as of H-7 to C-5, C-6, C-8, and Me-21 in the HMBC spectrum (Figure 2). Thus, the planar structure of compound **2** was determined.

Compound **3** was isolated as a pale yellow amorphous solid and assigned the molecular formula of C_25_H_36_O_4_ according to the following positive HRESIMS *m/z* value: 423.2511 [M + Na]^+^ (calcd for 423.2506). Analysis of the ^1^H and ^13^C NMR data revealed that **3** (Table 1 and Table 2) was found to contain an additional ketone carbon when compared with those of terpestacin [9,10,11]. The key HMBC correlations of Me-22 to C-11, C-12, and C-13, as well as of H-9 to C-11 and H-10 to C-11, indicated the presence of an additional ketone carbonyl in C-11 (Figure 2); this was also indicated through comparison of the data with those that were in agreement with the data of terpestacin.

Compound **4** was isolated as a pale yellow amorphous solid. The HRESIMS of **4** established its molecular formula as C_25_H_38_O_5_, thus indicating seven degrees of unsaturation. The ^1^H and ^13^C NMR spectra of **4** (Table 1 and Table 2) showed a close similarity to those of terpestacin, [9,10,11] with the exception that one trisubstituted double bond of the latter was replaced by an epoxy three-membered ring (*δ*_C_ of 63.8 and 62.0, respectively), which was confirmed by the HRESIMS data. This assumption was supported by the correlation of Me-22 to C-11, C-12, and C-13, as well as of H-13 to C-12, C-14, and C-15 in the HMBC spectrum (Figure 2). Accordingly, the planar structure of **4** was constructed, as shown in Figure 1.

**Figure 3:**
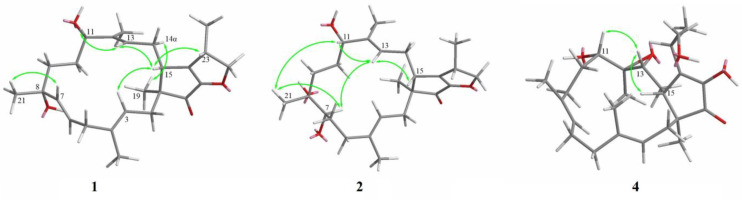
Key NOESY correlations of compounds **1**, **2**, and **4**.

**Figure 4:**
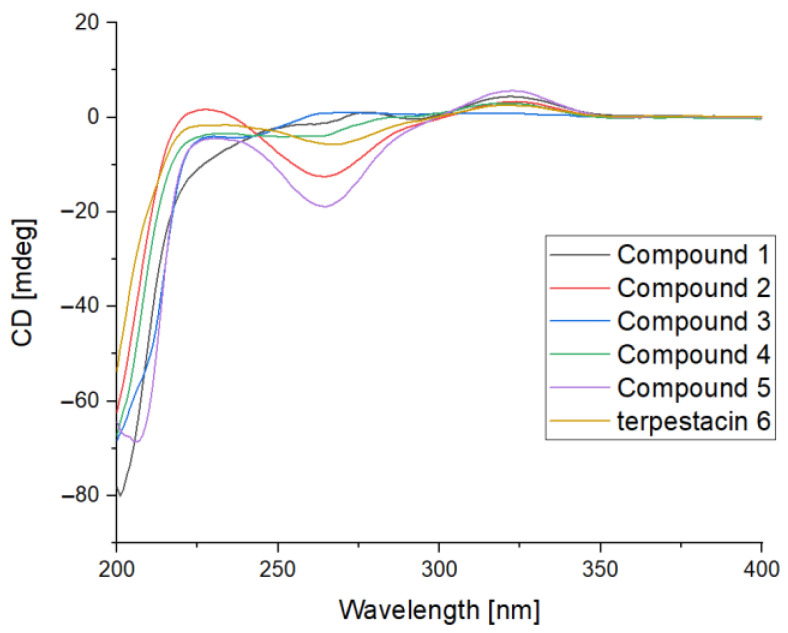
Experimental ECD spectrum of **1**−**6.**

The similar relative configurations of compounds **1**−**4** were determined through analyses of the coupling constants and through NOESY experiments, as well as through a comparison with the data of terpestacin (**6**), which were originally determined using X-ray crystallography. A strong NOE was seen between Me-19 and H-14α, and the latter was not coupled with H-15, which are consistent with the literature concerning the *trans* relationship of 5- and 15-membered rings (Figure 3) [10,14,28]. In the NOESY spectrum, the correlations of H-15 to H-3, H-13, and H-23, and of H-11 to H-13 revealed the *β*-configurations of these H atoms. The *E*-geometry of *∆*3, *∆*7, and *∆*12 were deduced from the *δ*_C_ values of Me-20, Me-21, and Me-22 (<20 ppm; Table 2) [10]. The configuration of *∆*6 in **1** was deduced as *E* as a result of using a large coupling constant of *J*_6,7_ = 15.4 Hz. The NOESY correlations of H-7 to H-13, H-21 to H-11 and H-7, and H-13 to H-15 in **2** deduced from H-7, H-21, H-11, and H-15 were on the same plane (Figure 3). In the NOESY spectrum of **4**, the correlations of H-13 to H-15 and H-13 to H-11 deduced from H-11, H-13, and H-15 were on the same plane (Figure 3). The same relative configurations, similar to the CD spectrum and similar specific options of the terpestacin derivatives, together with the same biogenetic origin, indicated that they also shared an identical absolute configuration (Figure 4).

By comparing the ^1^H, ^13^C-NMR, and MS data with the literature values, the known compounds were identified as 21-hydroxyterpestacin (**5**) [28], terpestacin (**6**) [9,10,11], fusaproliferin (**7**) [13], and saponaroxin A (**8**) [27]. Based on our isolated terpestacin, seventeen derivatives with various L-amino acid side chains were designed and synthesized. Their anticancer activities against U87MG-derived glioblastoma stem cells (GSCs) were evaluated, and two derivatives showed stronger activities related to their further development as anticancer agents for the treatment of GBM by targeting the GSCs [17].

**Figure 5:**
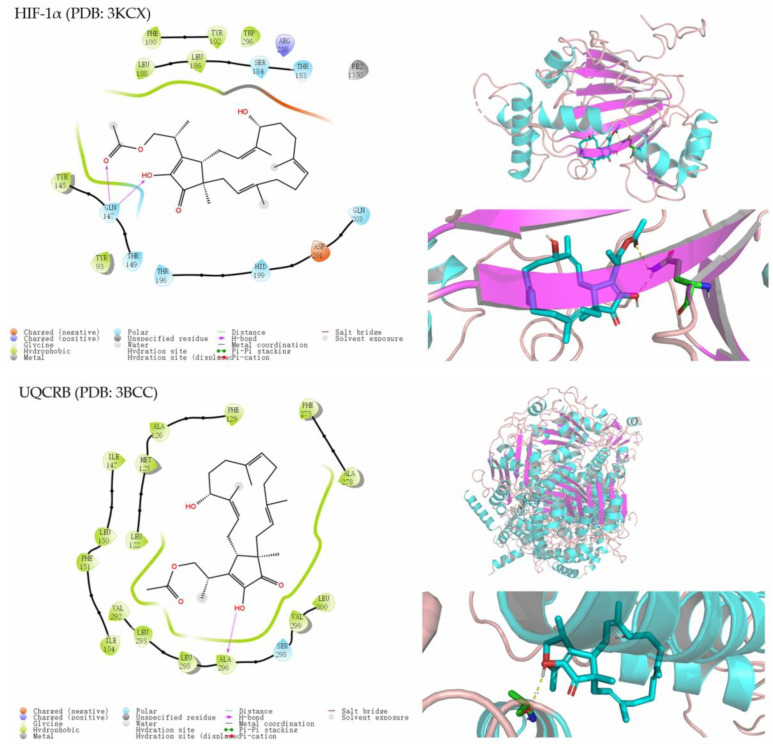
The 2D and 3D models of **7** with HIF-1α (PDB: 3KCX) and UQCRB (PDB: 3BCC) predicted using in silico molecular docking.

All of the compounds were evaluated for their cytotoxic activities against the human cancer cells MDAMB-231, C4-2B, MGC803, MDA-MB-468, and A549 using the CCK-8 method, but they exhibited no inhibitory activities at 5 μM. The primary screening of compounds **2**, **5**, and **6** regarding the proliferation of U87MG-derived GSCs was carried out using an ATP-monitoring luminescence assay [17]. The results revealed that terpestacin (**6**) exhibited significant activity, with an IC_50_ value of 20.3 μM, compared to the positive control A1938, with an IC_50_ value of 10.9 μM. Additionally, compounds **2** and **5** were found to show weak inhibitory effects. Furthermore, the levels of hypoxia-inducible Factor-1α (HIF-1α) and CD133 protein expression levels were evaluated using in vitro experiments after the treatment of the compounds **2**, **5**, and **6**. Compared to terpestacin and the positive control 4-200, compound **5** showed stronger activity (Figure S26). Because of the diverse biological activities that have been reported regarding terpestacin [29,30], molecular docking studies of these isolated compounds were carried out in silico with HIF-1α (PDB: 3KCX) and UQCRB (PDB: 3BCC) active sites to further understand the binding mode between those derivatives with the protein. As a result, compounds **2**, **5**, and **6** appeared to interact with the HIF-1α protein with docking scores of −4.284, −5.875, and −4.565, respectively, as well as with the UQCRB protein with docking scores of −5.820, −8.023, and −7.425, respectively, according to the above experiment data (Figure 5). Although the in vitro tests of **7** were limited by the small amounts, the similar interactions in the 2D binding models of the HIF-1α and UQCRB—with better docking scores of −5.875 and −8.886, respectively—suggested that **7** may act as a potential anticancer agent against U87MG-derived GSCs like its analogs. Compound **7** interacted with the HIF-1α active site mainly through hydrogen bonds between the Gln14 of the protein 3KCX, as well as with the ester groups and hydroxy. The OH-17 of **7** formed hydrogen bonds with the Ala296 of protein 3BCC. These results provide valuable information for the further development of anticancer agents.

## 3. Materials and Methods

### 3.1. General Experimental Procedures

Optical rotation values were achieved using an Anton Paar MCP-500 polarimeter (Anton, Austria). The one-dimensional and two-dimensional (2D) nuclear magnetic resonance (NMR) spectra were obtained using a Bruker Avance spectrometer (AC 500 or AVANCE III HD 700 NMR) with tetramethylsilane (TMS) as an internal standard. High-resolution electrospray ionization mass spectrometry (HR-ESI-MS) data were measured using a Bruker microTOF-QII mass spectrometer (Bruker, Fällanden, Switzerland). CD spectra were measured using a Chirascan circular dichroism spectrometer (Applied Photophysics). YMC gel (ODS-A, 12 nm, S-50 µm) and silica gel (200–300 mesh) (Qingdao Marine Chemical Factory) were used for column chromatography. The silica gel GF254 (Qingdao Marine Chemical Factory, Qingdao, China) was used for TLC. A semipreparative HPLC was performed using a Hitachi L-2400 (diode array detector, Hitachi L-2455, Tokyo, Japan) using a YMC ODS column (YMC-pack ODS-A, 10 × 250 mm, 5 mm, YMC Co. Ltd., Kyoto, Japan). All solvents used were of analytical grade (Tianjin Fuyu Chemical and Industry Factory). Spots were detected on TLC under UV light or by heating after spraying with 5% H_2_SO_4_ in EtOH (*v*/*v*).

### 3.2. Fungal Material

The culture of *Arthrinium* sp. SCSIO41221 was isolated from a mangrove sediment sample collected in Sanya (18°13′50.2″ N, 109°37′15.8″ E) in August 2010. The internal transcribed spacer (ITS) sequences of SCSIO41221 shared a similarity of 99% to that of *Arthrinium sp.* LH11 (GenBank accession no. HQ832842.1). On the basis of its molecular biological protocol and morphological analyses, strain SCSIO 41221 was identified as *Arthrinium* sp. and designated as *Arthrinium* sp. SCSIO 41221. The strain was stored on PDA slants at 4 °C and deposited in the RNAM Center.

### 3.3. Extraction and Isolation

The fungus was inoculated in PDA agar medium (infusion of 200 g of potato, 20 g of dextrose, 2.5 g of NaCl, and 1000 mL of distilled water) as the seed medium and incubated at 25 °C on a rotating shaker (180 rpm) for 2 days. Then, 10 mL seed solution were inoculated in rice medium (200 g of rice, 0.5 g of NaCl, and 200 mL of distilled water) in 1000 mL Erlenmeyer flasks. The mass fermentation of this fungus was carried out at 25 °C under static conditions for 50 days. After 50 days, the fermentation was cut into small pieces, sonicated for 20 min, and soaked in acetone three times. The acetone extract was concentrated under reduced pressure to afford an aqueous solution, and then the aqueous solution was extracted using EtOAc to gain 51 g of a crude gum.

The crude extract was subjected to silica gel column chromatography, which was eluted with CHCl_3_–MeOH mixed solvent in a step gradient to give twenty-seven fractions (Fr-1~27). Fr-10 was applied to ODS gel column chromatography using medium pressure liquid chromatography (MPLC) eluted with a gradient of MeOH/H_2_O (1:9–1:0, *v*/*v*), and it was further purified using semipreparative RP HPLC (73% MeOH in H_2_O, 3 mL/min) to afford **1** (2.1 mg, t_R_ = 13.1 min), **7** (1.7 mg, *t*_R_ = 17.3 min), and **8** (1.5 mg, *t*_R_ = 21.5 min). Frs.13 was subjected to ODS gel column chromatography using MPLC with a gradient of MeOH/H_2_O (1:9–1:0, *v*/*v*), and it was purified using semipreparative RP HPLC (65% MeOH in H_2_O, 3 mL/min) to afford **3** (1.6 mg, *t*_R_ = 41.0 min). Frs. 18 was subjected to ODS gel column chromatography using a gradient of MeOH/H_2_O (1:9–1:0, *v*/*v*), to yield 16 fractions (Frs. 18-1~18-16). Frs. 18-6 was purified using semipreparative RP HPLC (45% MeOH in H_2_O, 3 mL/min) to afford **6** (210 mg, *t*_R_ = 38.8 min). Frs. 18-8 was purified using semipreparative RP HPLC (60% MeOH in H_2_O, 3 mL/min) to afford **2** (12.2 mg, *t*_R_ = 28.0 min). Frs. 18-9 was purified using semipreparative RP HPLC (65% MeOH in H_2_O, 3 mL/min) to afford **4** (2.0 mg, *t*_R_ = 36.9 min). Frs. 27 was subjected to ODS gel column chromatography with MeOH/H_2_O (1:9–1:0, *v*/*v*) and then was purified using semipreparative RP HPLC (45% MeOH in H_2_O, 3 mL/min) to afford **5** (95.5 mg, *t*_R_ = 38.8 min).

### 3.4. Compound Characterization

*Arthproliferin A (***1***)*: Pale yellow amorphous solid; [*α*${]}_{D}^{25}$ + 5.9 (MeOH; *c* 0.08); CD cm^2^mol^−1^: Δ*ε* 323 +2.72, Δ*ε* 201 −48.51 (MeOH; *c* 0.5); ^1^H and ^13^C NMR data: see Table 1 and Table 2; HRESIMS in *m/z*: 423.2516 [M + Na]^+^ (calcd for C_25_H_36_N_a_O_4_ 423.2506), *m/z*: 823.5128 [2M + Na]^+^ (calcd for C_50_H_72_NaO_8_ 823.5119).
*Arthproliferin B (***2***)*: Pale yellow oil; [*α*${]}_{D}^{25}$ + 33.5 (MeOH; *c* 1.2); CD cm^2^mol^−1^: Δ*ε* 324 +2.13, Δ*ε* 264 −7.91, Δ*ε* 200 −39.28 (MeOH; *c* 0.47); ^1^H and ^13^C NMR data: see Table 1 and Table 2; HRESIMS in *m/z*: 441.2623 [M + Na]^+^ (calcd for C_25_H_38_N_a_O_5_ 441.2611), *m/z*: 859.5351 [2M + Na]^+^ (calcd for C_50_H_76_NaO_10_ 859.5331).
*Arthproliferin C (***3***)*: Pale yellow amorphous solid; [*α*${]}_{D}^{25}$ − 62.8 (MeOH; *c* 0.29); CD cm^2^mol^−1^: Δ*ε* 238 −2.59, Δ*ε* 200 −41.24 (MeOH; *c* 0.47); ^1^H and ^13^C NMR data: see Table 1 and Table 2; HRESIMS in *m/z*: 423.2511 [M + Na]^+^ (calcd for C_25_H_36_N_a_O_4_ 423.2506), *m/z*: 823.5124 [2M + Na]^+^ (calcd for C_50_H_72_NaO_8_ 823.5119).
*Arthproliferin D (***4***)*: Pale yellow amorphous solid; [*α*${]}_{D}^{25}$ + 4.9 (MeOH; *c* 0.05); CD cm^2^mol^−1^: Δ*ε* 322 +1.88, Δ*ε* 262 −2.53, Δ*ε* 200 −42.46 (MeOH; *c* 0.47); ^1^H and ^13^C NMR data: see Table 1 and Table 2; HRESIMS in *m/z*: 441.2617 [M + Na]^+^ (calcd for C_25_H_38_N_a_O_5_ 441.2611), *m/z*: 859.5332 [2M + Na]^+^ (calcd for C_50_H_76_NaO_10_ 859.5331).

### 3.5. Bioassay

Cytotoxicity was assayed using the CCK-8 method, which was previously described [31]. Five human cancer cell lines (human breast cancer cells MDAMB-231, human breast cancer cells C4-2B, human gastric cancer cells MGC803, human breast cancer cells MDA-MB-468, and human breast cancer cells A549) were used in the cytotoxic activity assay. These cell lines were obtained from the National Infrastructure of Cell Line Resource (NICR) and Shanghai Cell Bank through the Chinese Academy of Sciences. Taxol was used as a positive control.

A preliminary screening of compounds **1**–**8** on the proliferation of U87MG-derived GSCs was determined using an ATP-monitoring luminescence assay as previously described [17]. A1938 was used as a positive control. The abilities of compounds **2**, **5**, and **6** to influence the expression of HIF-1α and CD133 were also investigated as previously described [17]. U87MG was obtained from the Korean Cell Line Bank (Seoul, Korea). Statistical analyses were performed using SPSS 9.0 software.

### 3.6. Molecular Docking Analysis

The molecular docking was conducted using the Schrödinger 2017-1 suite as described previously [32]. The crystal structures of human HIF-1α with Clioquinol inhibitor (PDB ID: 3KCX) and UQCRB (PDB ID: 3BCC) were collected from the protein data bank (http://www.pdb.org, accessed on 15 September 2023). The structures of these docking compounds were generated in ChemBio3D Ultra 20.0, followed by an MM2 calculation to minimize the conformation energy. The 3D structures of the binding models were generated using PyMol molecular graphics software (Maestro Version 11.1.011, MMshare Version 3.7.011, Release 2017-1, Platform Windows-x64) (Schrödinger 2017-1, Schrödinger Inc., New York, NY, USA). The XYZ coordinates of the protein 3KCK with the ligand were −20.95, 25.69, and 7.97, respectively. The XYZ coordinates of the protein 3BCC with the ligand were 8.78, 128.61, and 83.01, respectively.

## 4. Conclusions

In summary, the chemical investigation of the marine mangrove-sediment-derived fungus *Arthrinium* sp. SCSIO41221 led to the isolation of four new sesterterpenes—arthproliferins A–D (**1**–**4**)—and four known derivatives—21-hydroxyterpestacin (**5**), terpestacin (**6**), fusaproliferin (**7**), and saponaroxin A (**8**). Their planar structures and absolute configurations were elucidated using detailed NMR, MS spectroscopic analyses, and comparison of the coupling constants, as well as through NOESY experiments and determining the ECD spectra with terpestacin, which was determined using X-ray crystallography. Terpestacin has been proven to display attractive anti-HIV and anticancer activities [11,30,33]. It can also inhibit angiogenesis both in vitro and in vivo via the downregulation of the ROS/HIF1α/VEGF pathway [17,30]. All of these isolated terpestacin derivatives were evaluated with respect to their cytotoxic activities. Compounds **2** and **5** were found to show weak inhibitory effects against U87MG-derived GSCs compared to terpestacin, which exhibited significant activity with an IC_50_ value of 20.3 μM. Further related enzymatic bioassays were evaluated, and compound **5** showed stronger abilities than terpestacin in HIF-1α and CD133 protein degradation. Molecular docking with the HIF-1α and UQCRB proteins was also performed to understand the inhibitory activity.

## Figures and Tables

**Table 1:** ^1^H NMR data for **1**–**4** (TMS in *δ* ppm).

No.	1 ^a^	2 ^b^	3 ^c^	4 ^a^
2	2.32, dd (9.1, 14.0)	2.06, m	2.03 dd (7.0, 16.8)	2.41, dd (2.8, 13.3)
	1.78, m		1.78 dd (6.3, 13.3)	1.88, m
3	5.26, m	5.50, t (7.5)	5.15, t (7.7)	5.39, t (8.4)
5	2.72, dd (7.0, 11.2)	2.25 m	2.18, m	2.29, m
			1.89, t (11.2)	2.08, m
6	5.69, d (15.4)	2.10 m	2.13, m	2.30, m
		1.61, m	2.06, m	2.24, m
7	5.82, ddd (7.0, 11.2, 12.6)	3.60, d (9.5)	4.94, t (6.3)	5.14, t (6.3)
9	2.08, m	1.89, m	2.72, m	2.21, m
	1.81, m	1.78, m		2.04, m
10	2.26, m	2.24, m	2.31, m	1.78, m
	1.85, m	1.79, m	2.17, m	1.68, m
11	4.47, t (7.0)	4.46, d (7.5)		3.07, t (7.0)
13	5.68, m	5.61, t (5.5)	6.79, t (6.3)	2.91, dd (2.1, 7.7)
14	2.57, dd (7.0, 15.4)	2.51, d (14.0)	2.63. m	1.84, m
			1.97, ddd (8.4, 11.9, 15.4)	1.60, ddd (3.5, 9.45, 13.65)
15	2.46, d (9.8)	2.58, dd (2.0, 12.0)	2.76, dd (2.1, 11.2)	2.85, dd (2.8, 8.4)
19	1.01, s	1.06, s	0.91, s	1.04, s
20	1.69, s	1.71, s	1.55, s	1.70, s
21	1.34, s	1.15, s	1.58, s	1.65, s
22	1.67, s	1.55, s	1.71, s	1.27, s
23	2.63, q (7.0, 14.0)	2.63, q (7.0, 16.0)	2.60, q (7.0, 14.0)	2.73, q (7.0, 14.0)
24	3.86, dd (7.0, 10.5)	3.86, dd (7.0, 10.5)	3.63, dd (7.0, 10.5)	3.87, dd (7.0, 10.5)
	3.73, dd (7.0, 10.5)	3.73, dd (6.5, 10.5)	3.52, dd (7.0, 10.5)	3.74, dd (6.3, 10.5)
25	1.30, d (7.0)	1.26, d (7.0)	1.20, d (7.0)	1.27, d (7.0)

^a^ indicates recorded at 700 MHz in CD_3_OD. ^b^ indicates recorded at 500 MHz in CD_3_OD. ^c^ indicates recorded at 700 MHz in DMSO-*d*6.

**Table 2:** ^13^C NMR data for **1**–**4.**

No.	1 ^a^	2 ^b^	3 ^c^	4 ^a^
1	48.4, C	50.0, C	49.3, C	48.5, C
2	38.5, CH_2_	39.9, CH_2_	39.9, CH_2_	37.8, CH_2_
3	120.8, CH	120.6, CH	121.3, CH	121.1, CH
4	137.4, C	139.3, C	137.5, C	138.2, C
5	40.0, CH_2_	34.0, CH_2_	40.5, CH_2_	39.7, CH_2_
6	137.4, CH	31.0, CH_2_	23.8, CH_2_	23.4, CH_2_
7	126.2, CH	77.5, CH	122.0, CH	123.3, CH
8	83.1, C	87.4, C	134, C	133.4, C
9	35.2, CH_2_	36.4, CH_2_	34.6, CH_2_	33.3, CH_2_
10	30.6, CH_2_	30.4, CH_2_	34.0, CH_2_	30.2, CH_2_
11	82.4, CH	83.6, CH	201.6, C	75.3, CH
12	136.5, C	137.4, C	136.6, C	63.8, C
13	121.5, CH	123.5, CH	142.7, CH	62.0, CH
14	27.5, CH_2_	29.4, CH_2_	31.7, CH_2_	28.2, CH_2_
15	50.8, CH	50.8, CH	48.7, CH	47.5, CH
16	150.8, C	152.1, C	150.3, C	150.3, C
17	147.6, C	148.9, C	147.7, C	147.9, C
18	208.8, C	210.3, C	207.5, C	208.1, C
19	16.9, CH_3_	16.8, CH_3_	16.3, CH_3_	17.3, CH_3_
20	15.9, CH_3_	19.5, CH_3_	16.1, CH_3_	14.3, CH_3_
21	27.0, CH_3_	19.8, CH_3_	17.5, CH_3_	15.1, CH_3_
22	13.6, CH_3_	14.7, CH_3_	12.0, CH_3_	9.5, CH_3_
23	37.7, CH	38.9, CH	37.8, CH	37.0, CH
24	64.5, CH_2_	65.9, CH_2_	64.3, CH_2_	64.4, CH_2_
25	13.4, CH_3_	14.5, CH_3_	14.8, CH_3_	13.3, CH_3_

^a^ indicates recorded at 700 MHz in CD_3_OD. ^b^ indicates recorded at 500 MHz in CD_3_OD. ^c^ indicates recorded at 700 MHz in DMSO-*d*6.

## Data Availability

The research data are available in the Supporting Information.

## Supplementary Materials

The following supporting information can be downloaded at https://www.mdpi.com/article/10.3390/molecules28217246/s1: Figures S1–S24: The 1D and 2D NMR spectra, as well as HRESIMS data of compounds **1**–**4**; Figure S25: single-crystal X-ray structures of compound **6**; and Figure S26: effect of compounds **2**, **5**, and terpestacin (**6**) on the expression levels of HIF-1α and CD133 in U87MG-derived GSCs.
