# Supplementary material for: Arthproliferins A–D, Four New Sesterterpenes from the Mangrove-Sediment-Derived Fungus Arthrinium sp. SCSIO41221
Source: Molecules. 2023 Oct 24;28(21):7246. doi: 10.3390/molecules28217246 (PMC10648114; doi:10.3390/molecules28217246)

Supporting Information

Arthproliferins A–D, four new sesterterpenes from the mangrove sediment-derived fungus *Arthrinium* sp. SCSIO41221

Bin Yang [1,†,*], Cuitian Li [2,†], Ying Chen [1], Yanchun He [1], Jianglian She [1], Xuefeng Zhou [1], Huangming Tao [3], and Bo Peng [4,*]

[1] CAS Key Laboratory of Tropical Marine Bio-resources and Ecology / Guangdong Key Laboratory of Marine Materia Medica, South China Sea Institute of Oceanology, Chinese Academy of Sciences, Guangzhou 510301, China

[2] Marine Enviemental Engineering Center, South China Sea Institute of Oceanology, Chinese Academy of Sciences, Guangzhou 510515, China

[3] School of Traditional Chinese Medicine, Southern Medical University, Guangzhou 510515, P. R. China;

[4] Institute for Environmental and Climate Research, Jinan University, Guangzhou 511443, China

* Correspondence: yangbin@scsio.ac.cn (B.Y.); pengbo@jnu.edu.cn (B.P.)

[†] These authors contributed equally to this work.

List of supporting information

Figure S1: ^{1}H-NMR (700 MHz, CD_3OD) Spectrum of Compound **1**

Figure **S2:** ^{13}C-NMR (175 MHz, CD_3OD) Spectrum of Compound **1**

Figure **S3: H**MQC (700 MHz) Spectrum of Compound **1**

Figure **S4: H**MBC (700 MHz) Spectrum of Compound **1**

Figure **S5:** NOESY (700 MHz) Spectrum of Compound **1**

Figure **S6:** HRESI-MS Spectrum of Compound **1**

Figure **S7:** ^{1}H-NMR (500 MHz, CD_3OD) Spectrum of Compound **2**

Figure **S8:** ^{13}C-NMR (125 MHz, CD_3OD) Spectrum of Compound **2**

Figure **S9: H**MQC (500 MHz) Spectrum of Compound **2**

Figure **S10: H**MBC (500 MHz) Spectrum of Compound **2**

Figure **S11:** NOESY (500 MHz) Spectrum of Compound **2**

Figure **S12:** HRESI-MS Spectrum of Compound **2**

Figure **S13:** ^{1}H-NMR (700 MHz, DMSO-*d*6) Spectrum of Compound **3**

Figure **S14:** ^{13}C-NMR (175 MHz, DMSO-*d*6) Spectrum of Compound **3**

Figure **S15: H**MQC (700 MHz) Spectrum of Compound **3**

Figure **S16: H**MBC (700 MHz) Spectrum of Compound **3**

Figure **S17:** NOESY (700 MHz) Spectrum of Compound **3**

Figure **S18:** HRESI-MS Spectrum of Compound **3**

Figure S19: ^{1}H-NMR (700 MHz, CD_3OD) Spectrum of Compound **4**

Figure **S20:** ^{13}C-NMR (175 MHz, CD_3OD) Spectrum of Compound **4**

Figure **S21: H**MQC (700 MHz) Spectrum of Compound **4**

Figure **S22: H**MBC (700 MHz) Spectrum of Compound **4**

Figure **S23:** NOESY (700 MHz) Spectrum of Compound **4**

Figure **S24:** HRESI-MS Spectrum of Compound **4**

Figure S25: Single-crystal X-ray structures of Compound **6**

Figure S26: Effect of compounds **2**, **5**, and terpestacin (**6**) on the expression levels of HIF-1α and CD133 in U87MG-derived GSCs

Figure S1: ^{1}H-NMR (700 MHz, CD_3OD) Spectrum of Compound **1**

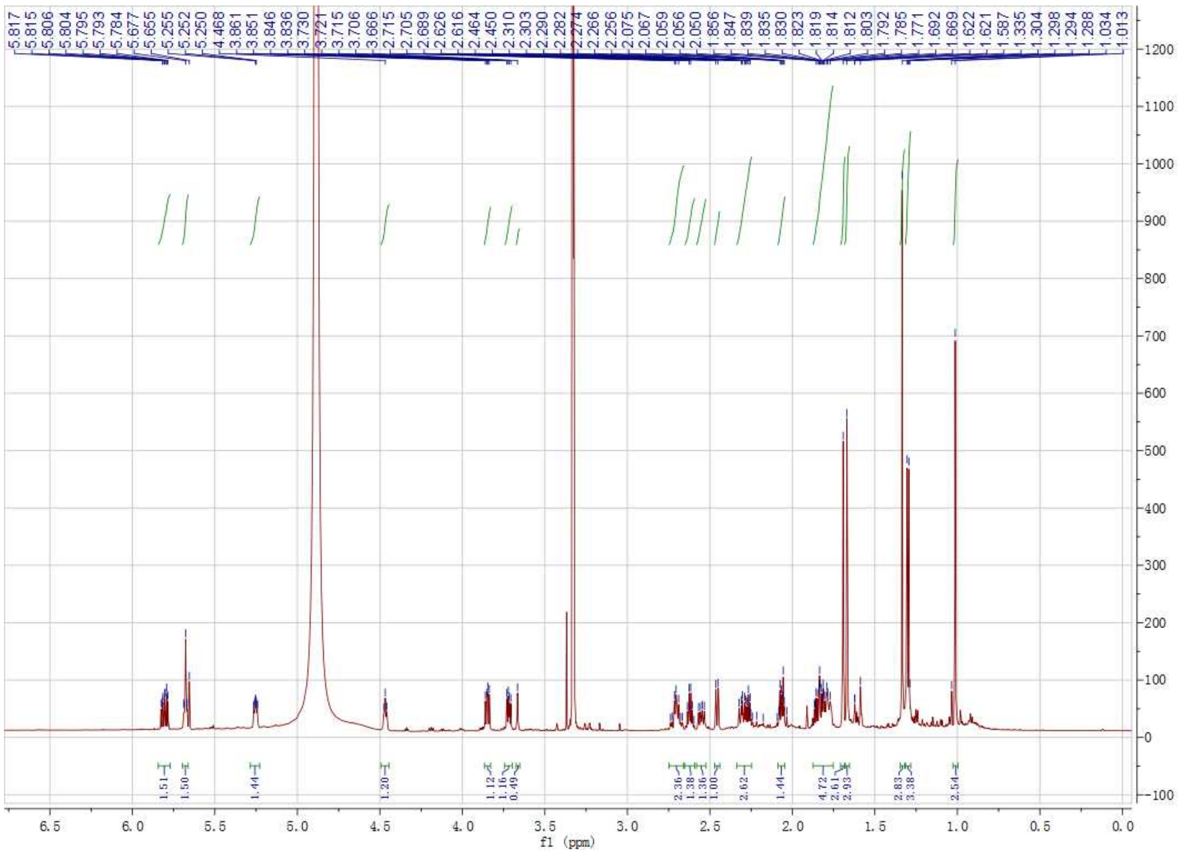

Figure S2: ^{13}C-NMR (175 MHz, CD_3OD) Spectrum of Compound **1**

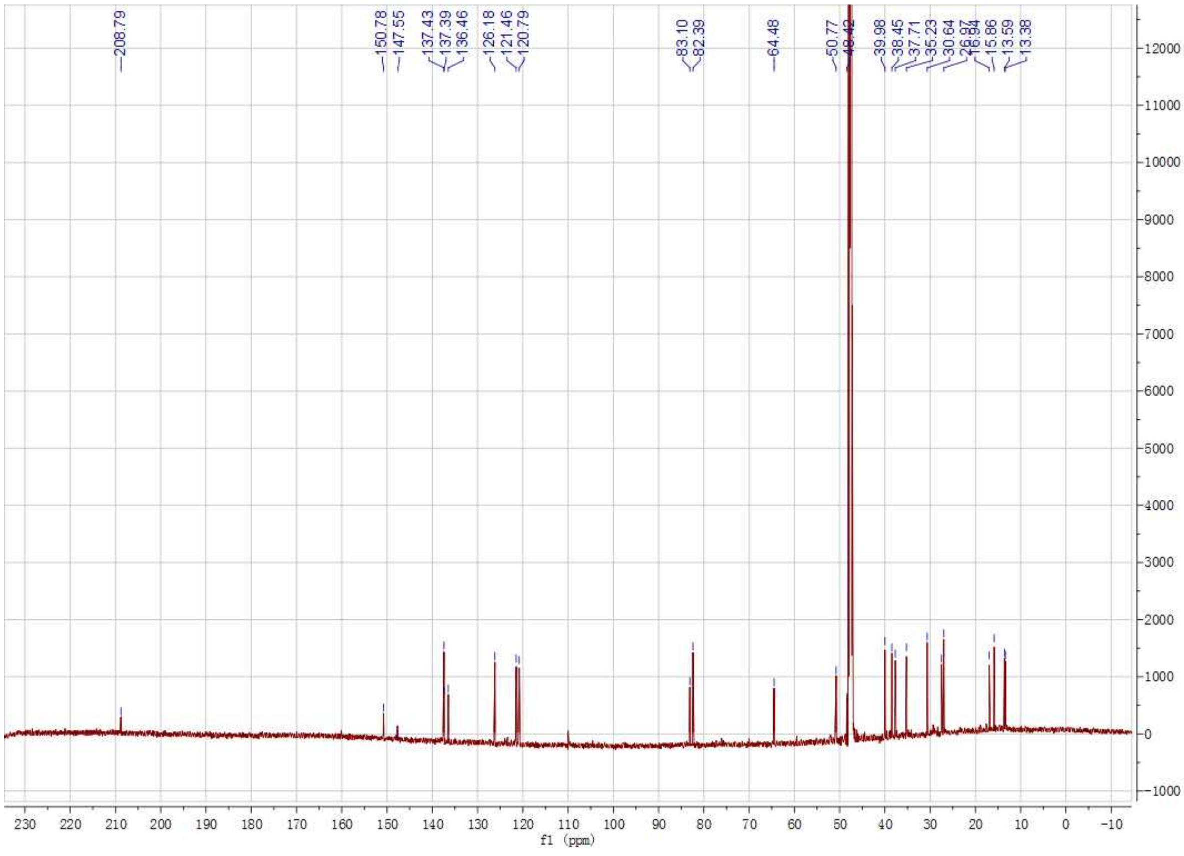

Figure S3: **H**MQC (700 MHz) Spectrum of Compound **1**

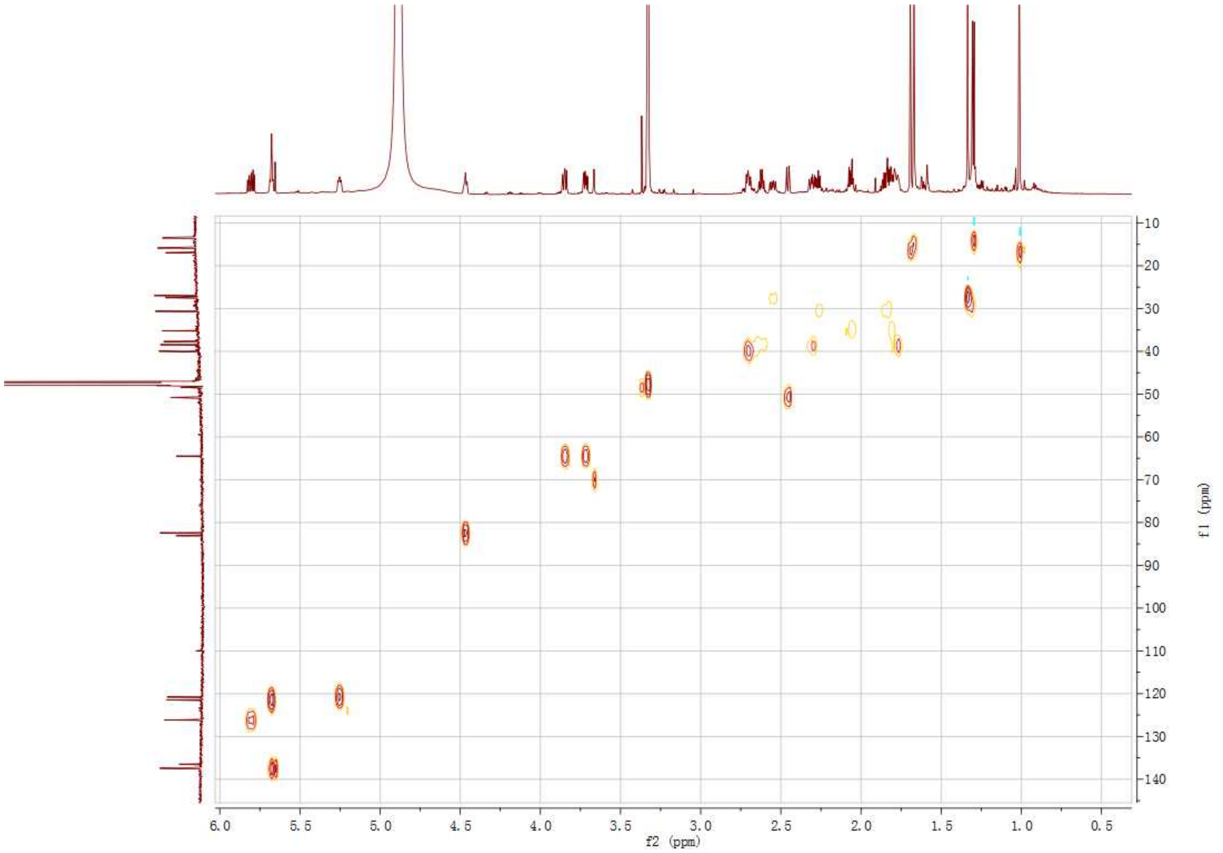

Figure S4: **H**MBC (700 MHz) Spectrum of Compound **1**

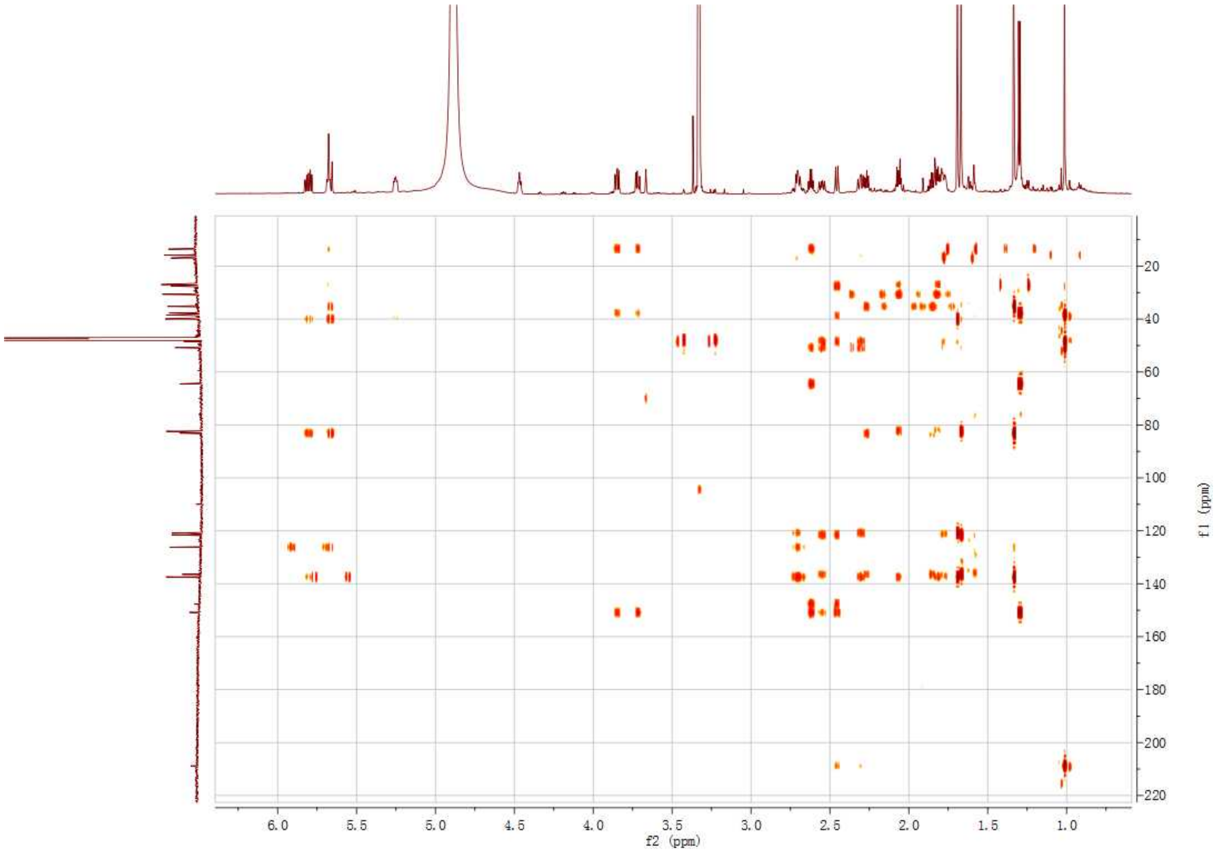

Figure S5: NOESY (700 MHz) Spectrum of Compound **1**

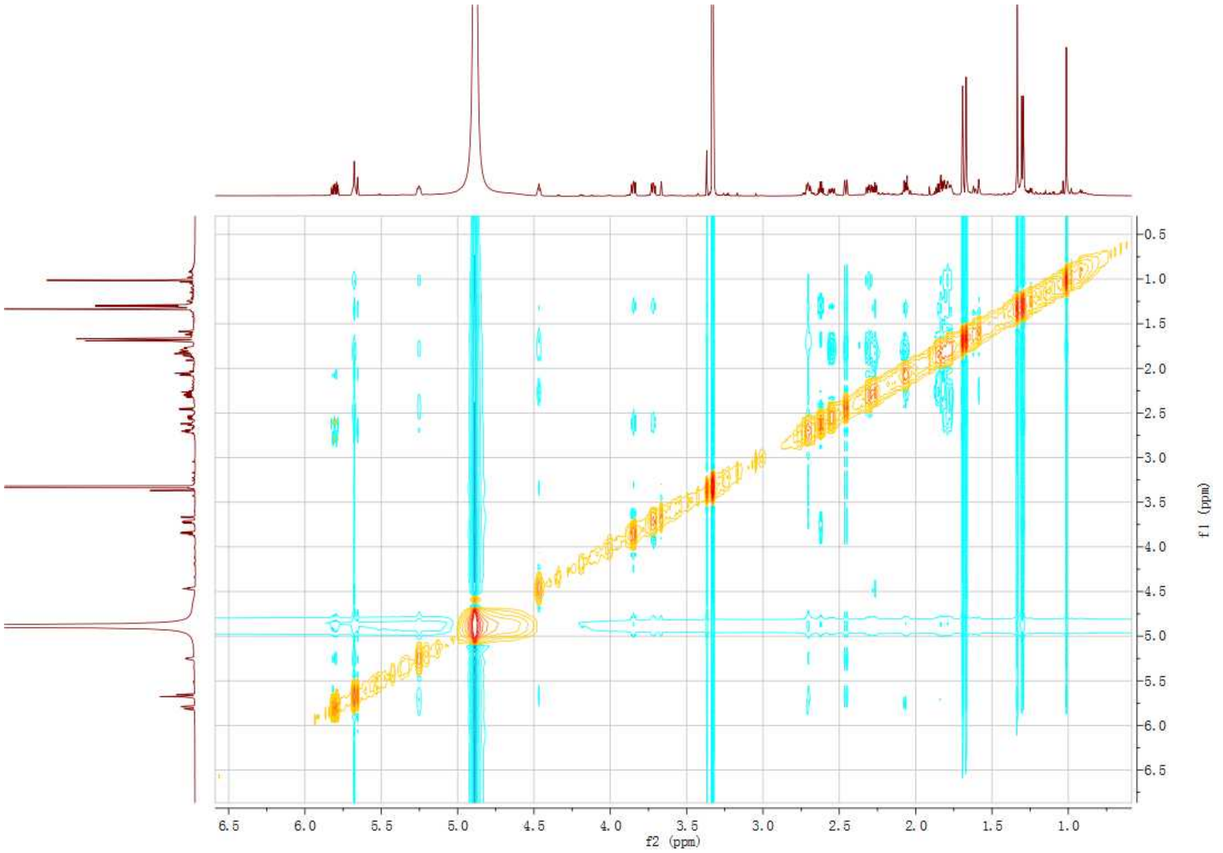

Figure S6: HRESI-MS Spectrum of Compound **1**

Mass Spectrum SmartFormula Report

Analysis Info		Acquisition	Date 6/12/2017 11:30:22 AM
Analysis Name	D:\Data\MS\data\201706\yangbin_YK4-1_pos_17_01_3111.d		
Method	LC_Direct Infusion_pos_70-500mz.m	Operator	SCSIO
Sample Name	yangbin_YK4-1_pos	Instrument	maXis 255552.00029
Comment			

Acquisition Parameter					
Source Type	ESI	Ion Polarity	Positive	Set Nebulizer	0.4 Bar
Focus	Active	Set Capillary	4500 V	Set Dry Heater	180 °C
Scan Begin	70 m/z	Set End Plate Offset	-500 V	Set Dry Gas	4.0 l/min
Scan End	1500 m/z	Set Charging Voltage	0 V	Set Divert Valve	Waste
		Set Corona	0 nA	Set APCI Heater	0 °C

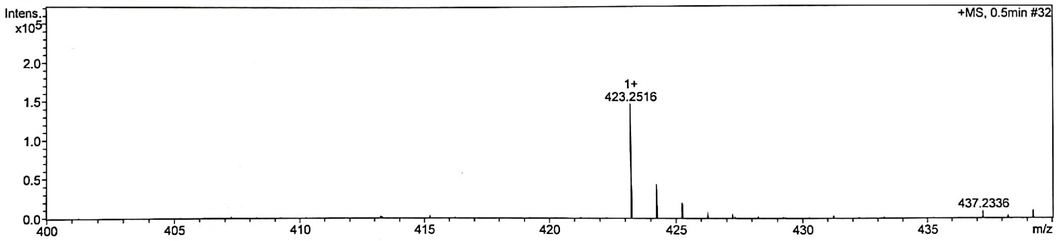

Meas. m/z	#	Ion Formula	Score	m/z	err [ppm]	err [mDa]	mSigma	rdb	e⁻ Conf	N-Rule
423.251604	1	C25H36NaO4	100.00	423.250580	-2.4	-1.0	48.0	7.5	even	ok
823.512849	1	C50H72NaO8	100.00	823.511940	1.1	0.9	118.2	14.5	even	ok

yangbin_YK4-1_pos_17_01_3111.d
Bruker Compass DataAnalysis 4.1 printed: 6/12/2017 11:35:43 AM by: SCSIO Page 1 of 1

Figure S7: ^{1}H-NMR (500 MHz, CD_3OD) Spectrum of Compound **2**

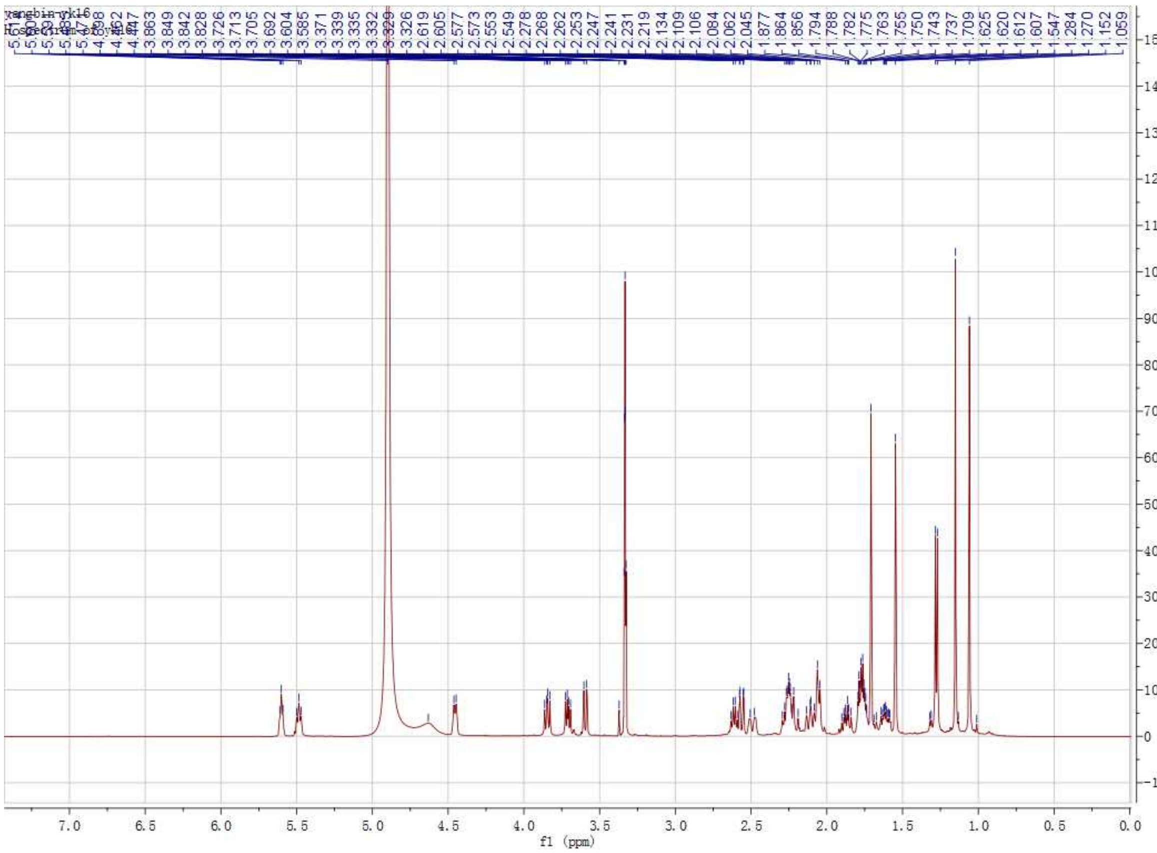

Figure S8: ^{13}C-NMR (125 MHz, CD_3OD) Spectrum of Compound **2**

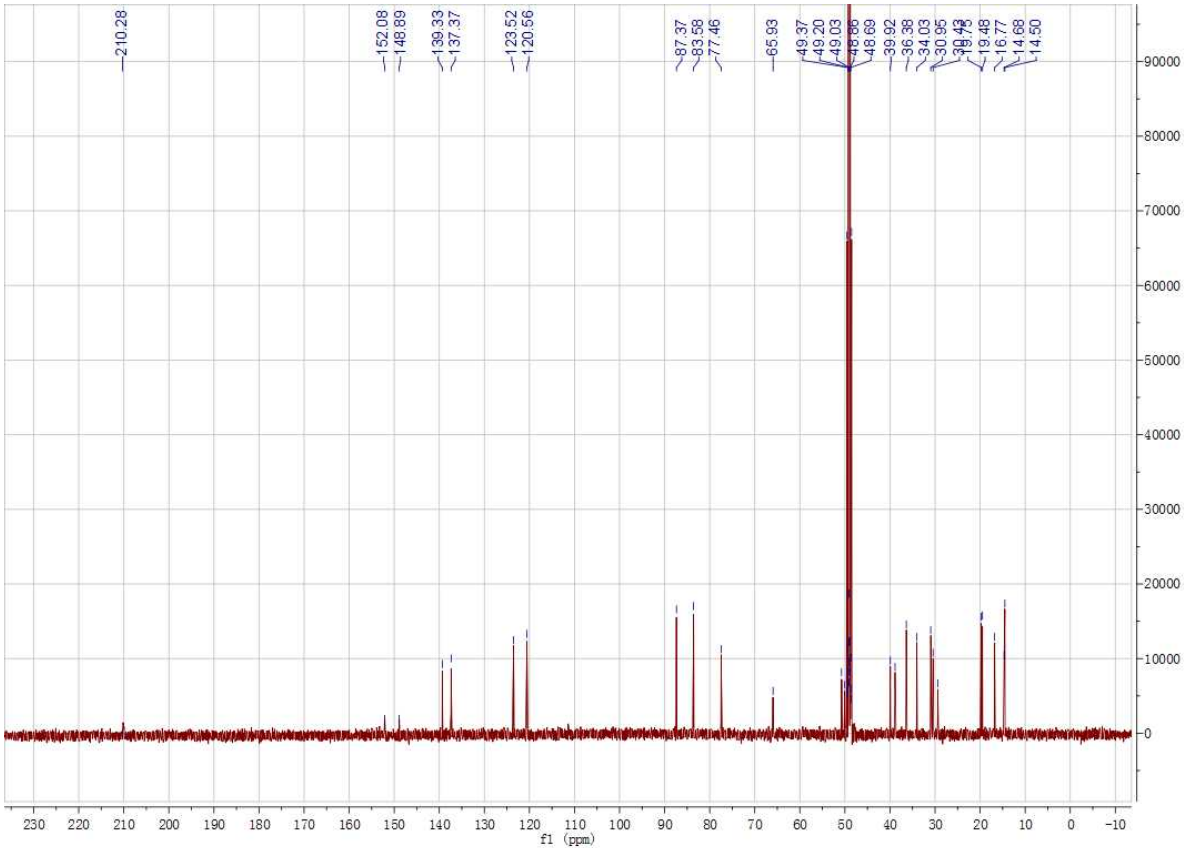

Figure S9: HMQC (500 MHz) Spectrum of Compound **2**

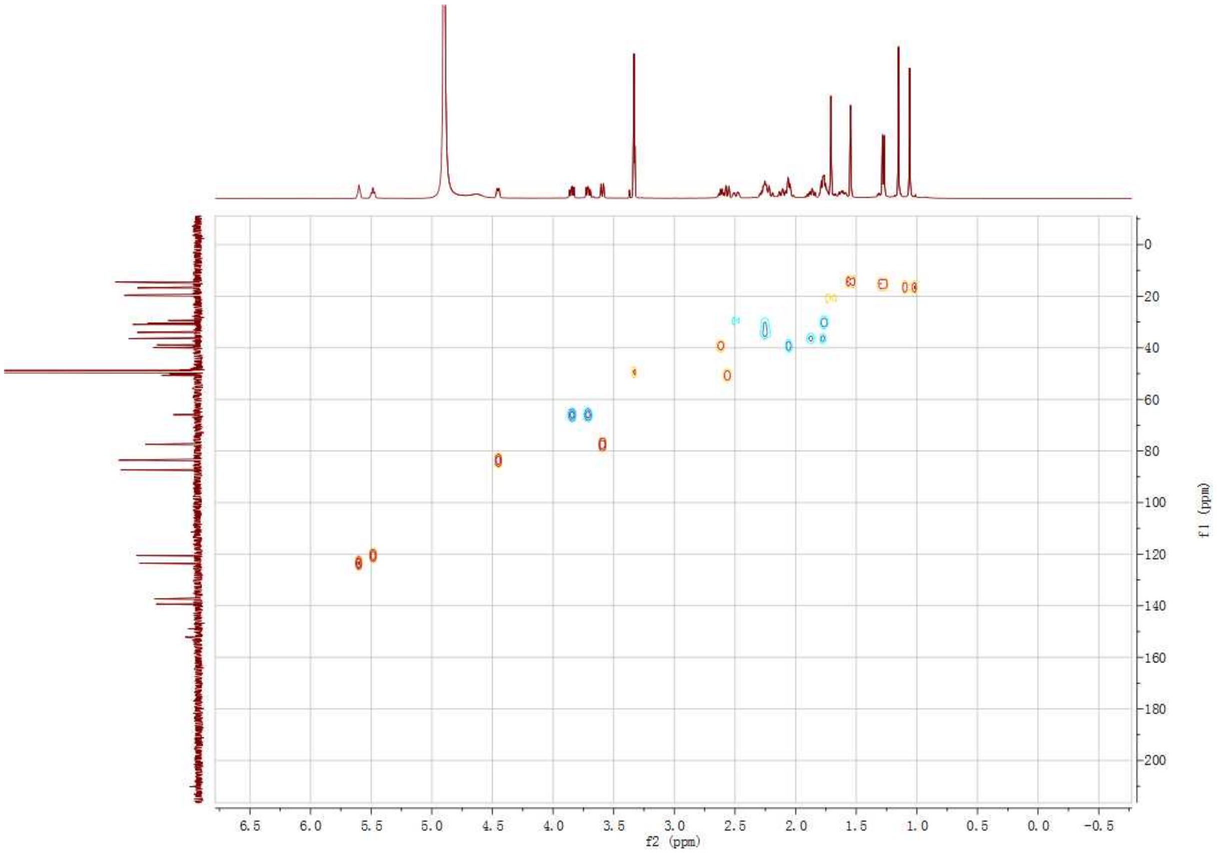

Figure S10: HMBC (500 MHz) Spectrum of Compound **2**

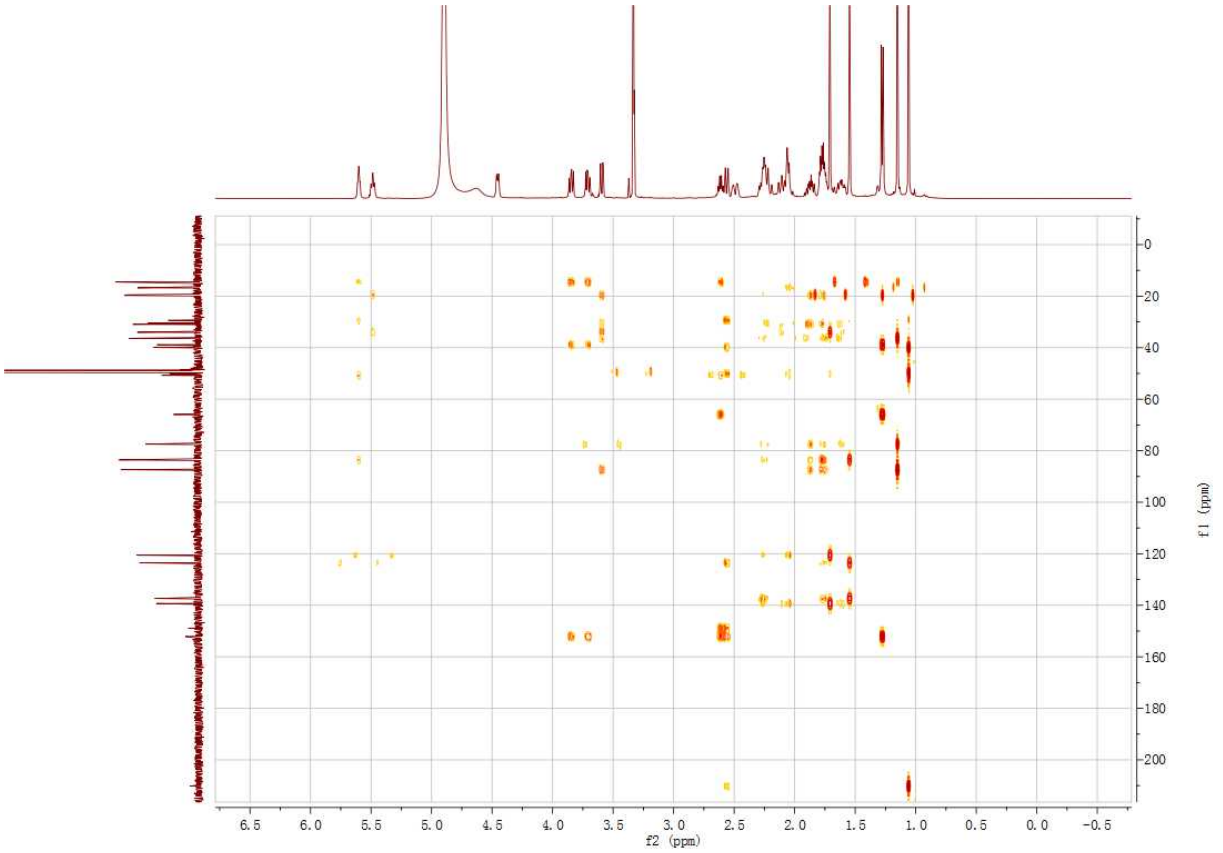

Figure S11: NOESY (500 MHz) Spectrum of Compound **2**

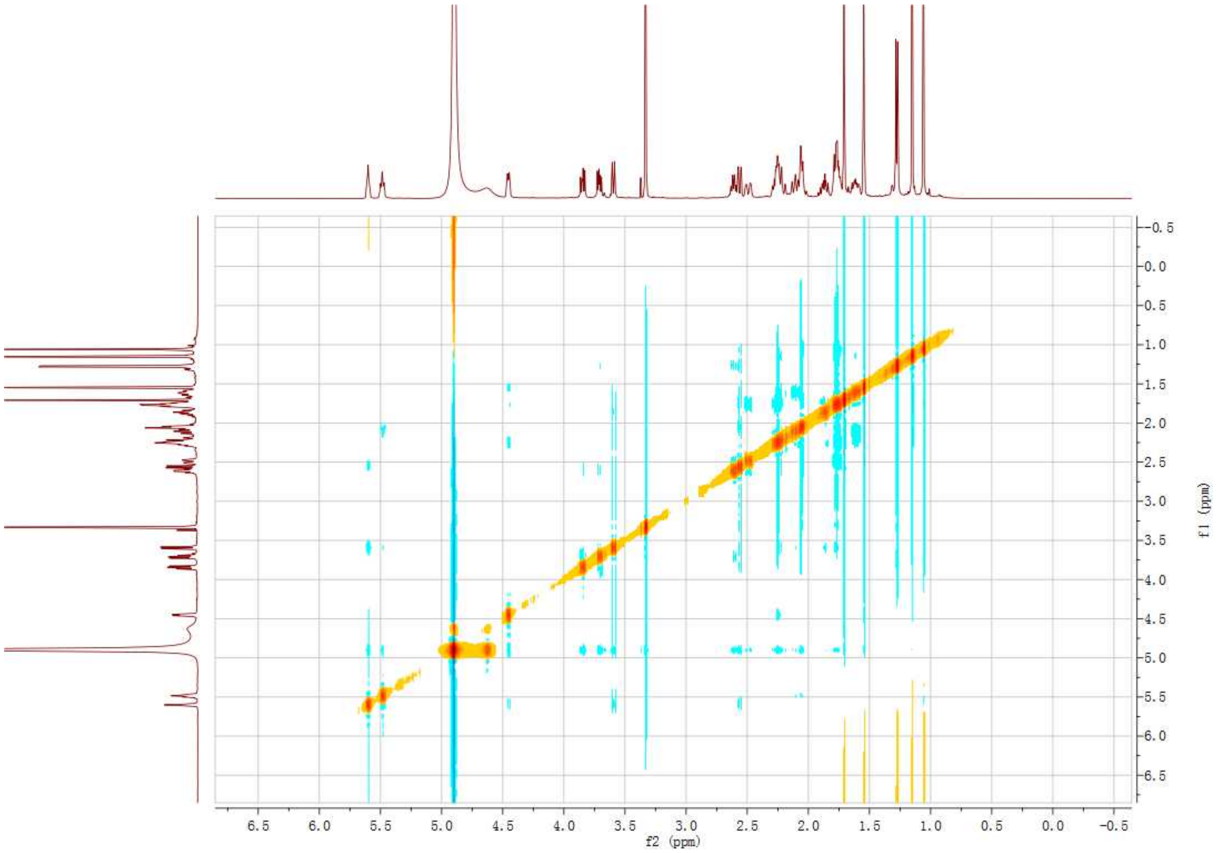

Figure S12: HRESI-MS Spectrum of Compound **2**

Mass Spectrum SmartFormula Report

**Analysis Info**		Acquisition Date	6/12/2017 11:16:32 AM
Analysis Name	D:\Data\MS\data\201706\yangbin_YK16_pos_13_01_3107.d		
Method	LC_Direct Infusion_pos_70-500mz.m	Operator	SCSIO
Sample Name	yangbin_YK16_pos	Instrument	maXis 255552.00029
Comment			

**Acquisition Parameter**					
Source Type	ESI	Ion Polarity	Positive	Set Nebulizer	0.4 Bar
Focus	Active	Set Capillary	4500 V	Set Dry Heater	180 °C
Scan Begin	70 m/z	Set End Plate Offset	-500 V	Set Dry Gas	4.0 l/min
Scan End	1500 m/z	Set Charging Voltage	0 V	Set Divert Valve	Waste
		Set Corona	0 nA	Set APCI Heater	0 °C

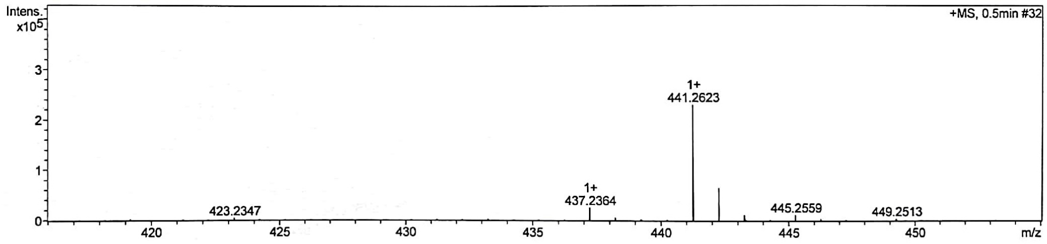

Meas. m/z	#	Ion Formula	Score	m/z	err [ppm]	err [mDa]	mSigma	rdb	e⁻ Conf	N-Rule
441.262285	1	C25H38NaO5	100.00	441.261145	-2.6	-1.1	3.8	6.5	even	ok
859.535121	1	C50H76NaO10	100.00	859.533069	-2.4	-2.1	61.5	12.5	even	ok

yangbin_YK16_pos_13_01_3107.d
Bruker Compass DataAnalysis 4.1 printed: 6/12/2017 11:45:24 AM by: SCSIO Page 1 of 1

Figure S13: ^{1}H-NMR (700 MHz, DMSO-*d6*) Spectrum of Compound **3**

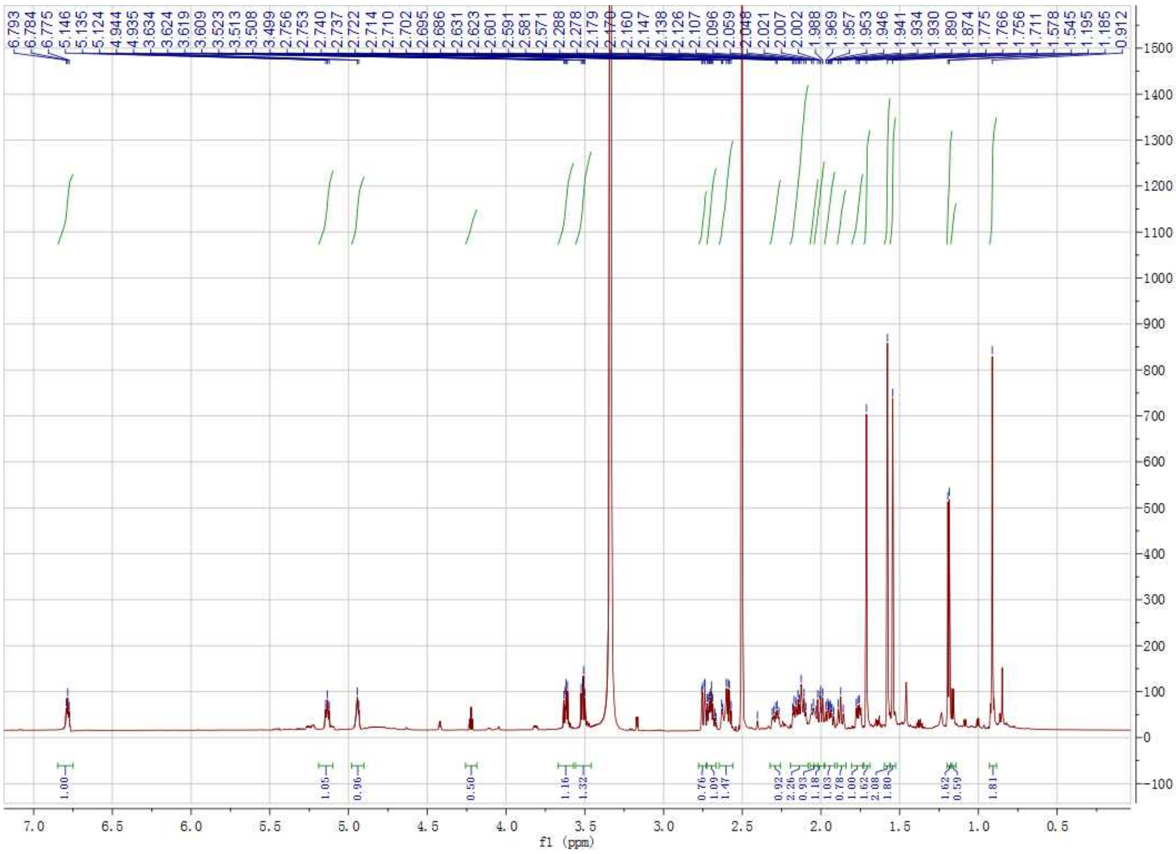

Figure S14: ^{13}C-NMR (175 MHz, DMSO-*d6*) Spectrum of Compound **3**

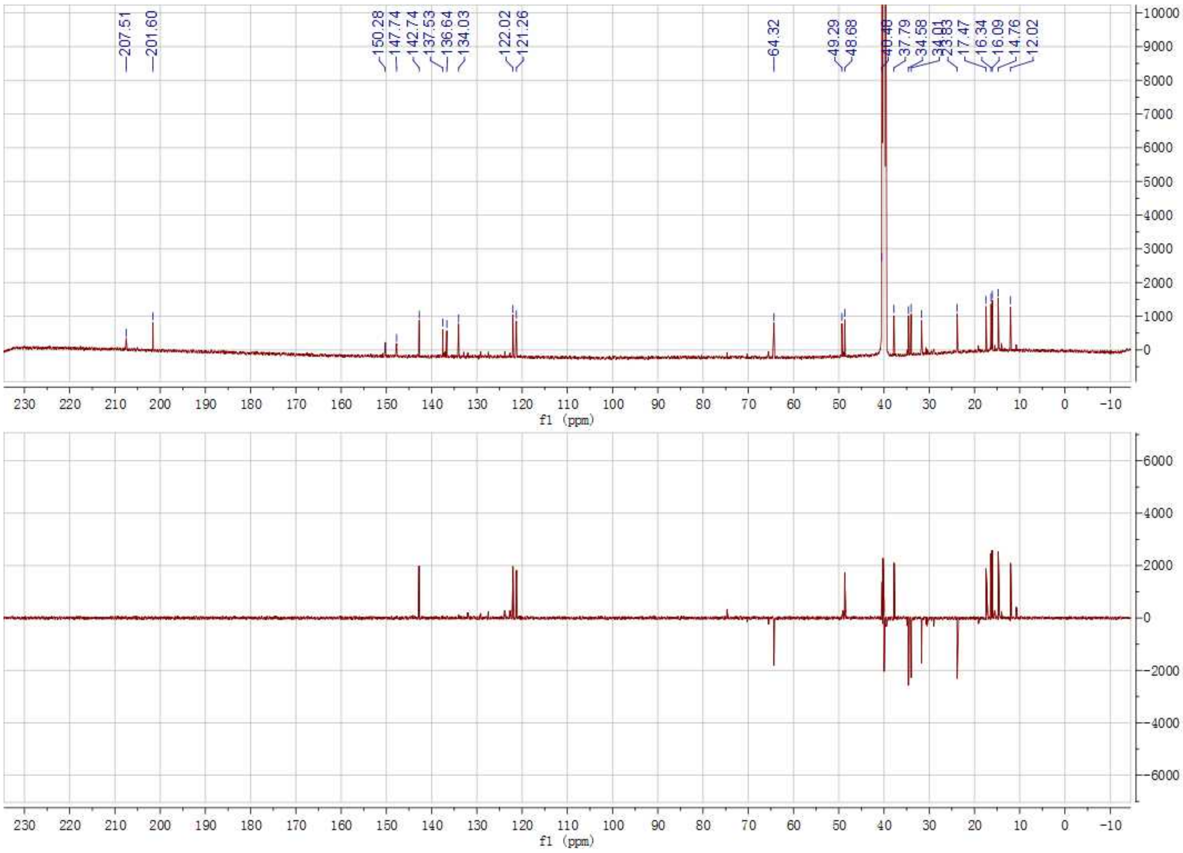

Figure S15: HMQC (700 MHz) Spectrum of Compound **3**

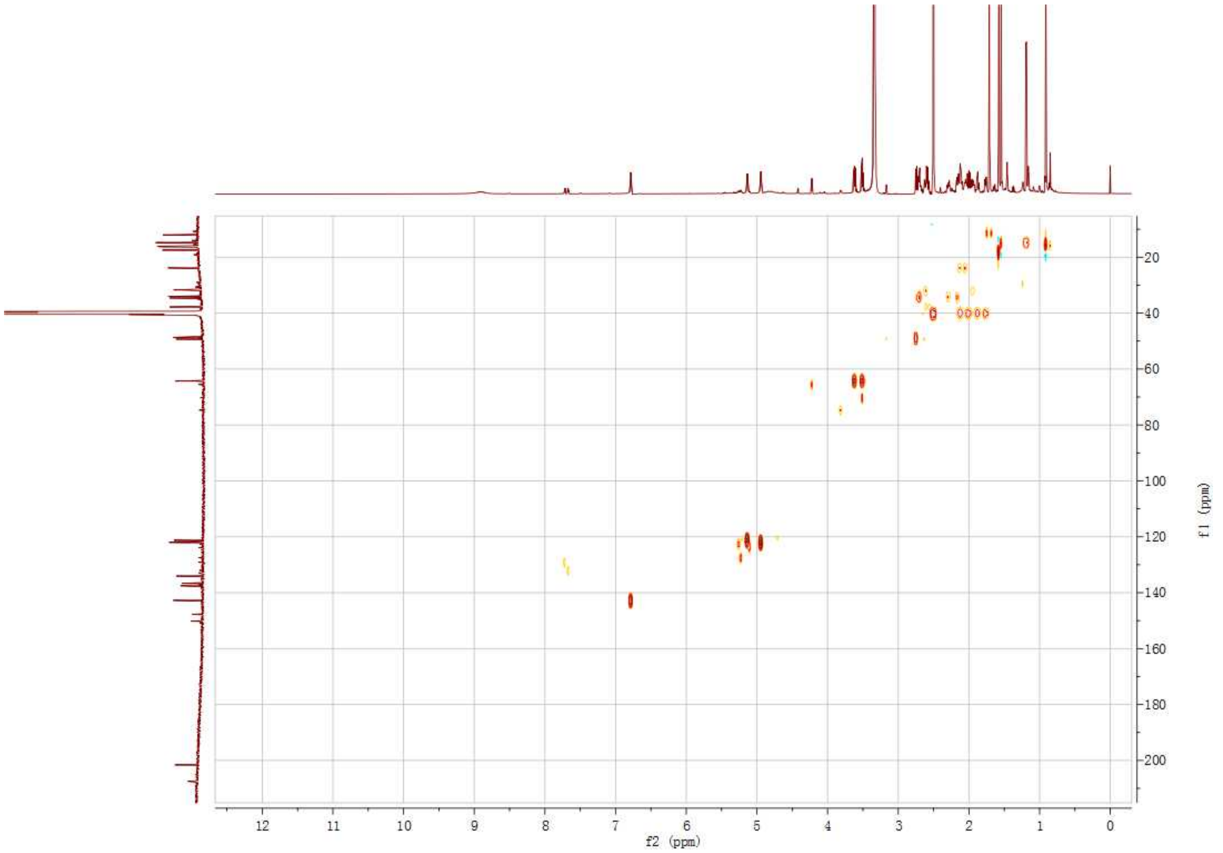

Figure S16: HMBC (700 MHz) Spectrum of Compound **3**

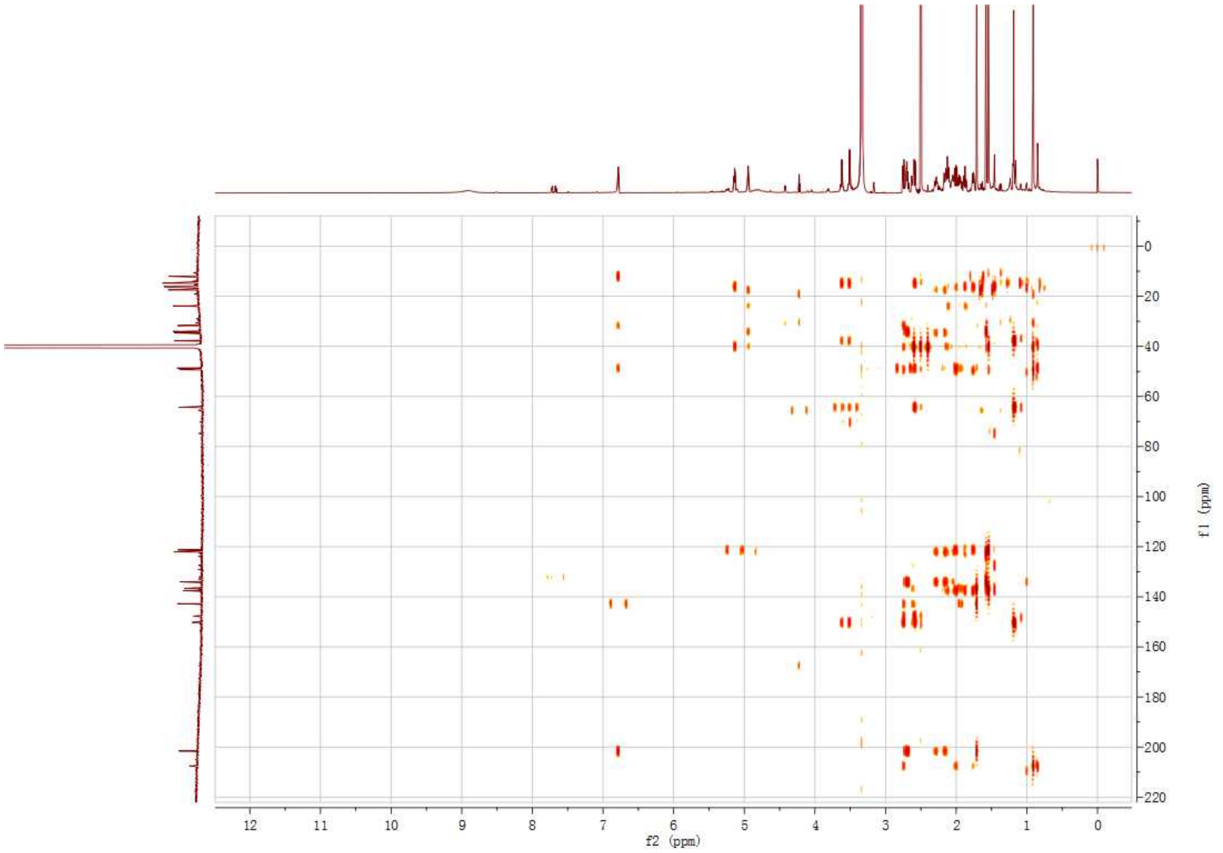

Figure S17: NOESY (700 MHz) Spectrum of Compound **3**

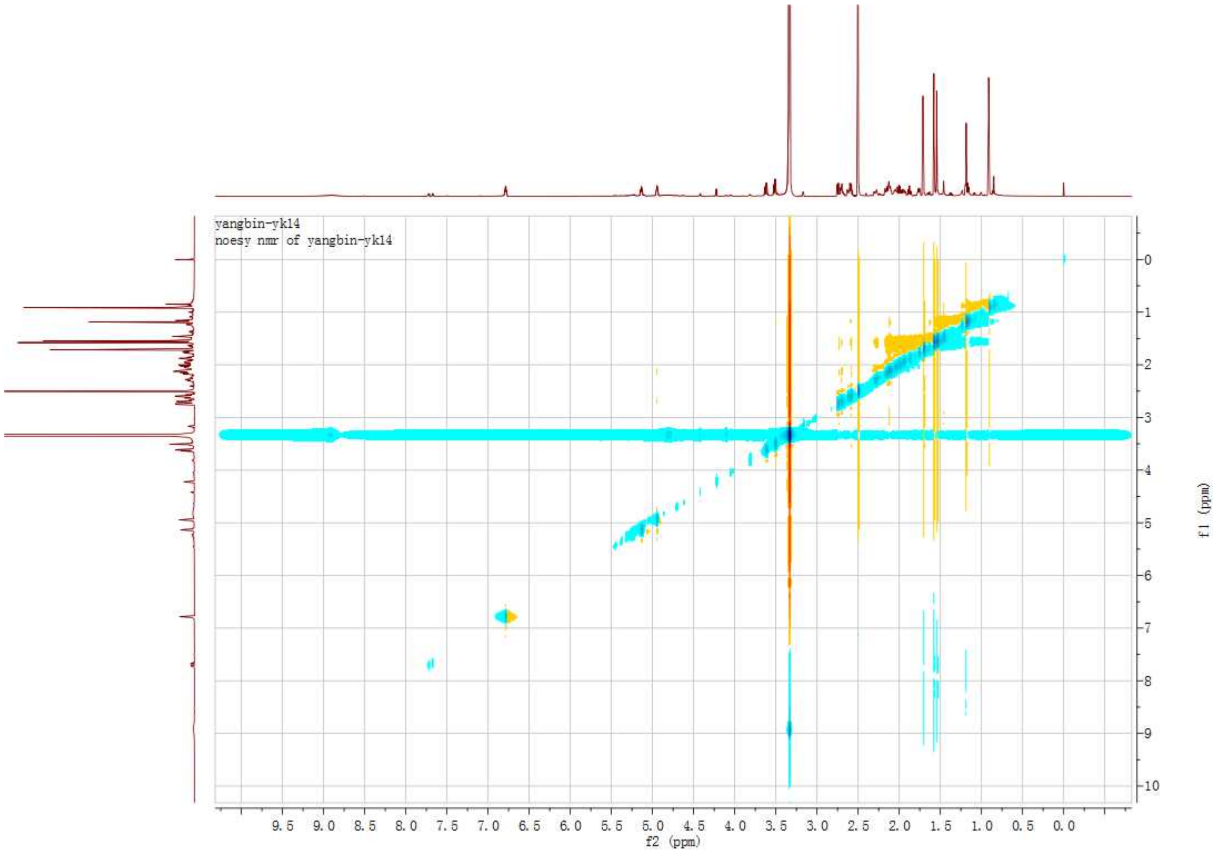

Figure S18: HRESI-MS Spectrum of Compound **3**

Mass Spectrum SmartFormula Report

**Analysis Info**		Acquisition Date	6/12/2017 11:13:07 AM
Analysis Name	D:\Data\MS\data\201706\yangbin_YK14_pos_12_01_3106.d		
Method	LC_Direct Infusion_pos_70-500mz.m	Operator	SCSIO
Sample Name	yangbin_YK14_pos	Instrument	maXis 255552.00029
Comment			

**Acquisition Parameter**					
Source Type	ESI	Ion Polarity	Positive	Set Nebulizer	0.4 Bar
Focus	Active	Set Capillary	4500 V	Set Dry Heater	180 °C
Scan Begin	70 m/z	Set End Plate Offset	-500 V	Set Dry Gas	4.0 l/min
Scan End	1500 m/z	Set Charging Voltage	0 V	Set Divert Valve	Waste
		Set Corona	0 nA	Set APCI Heater	0 °C

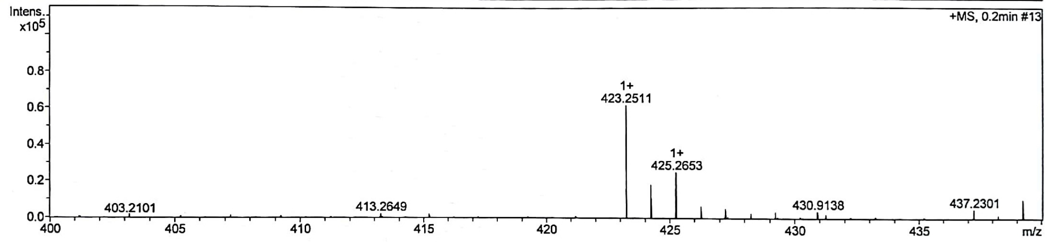

Meas. m/z	#	Ion Formula	Score	m/z	err [ppm]	err [mDa]	mSigma	rdb	e⁻ Conf	N-Rule
423.251105	1	C25H36NaO4	100.00	423.250580	1.2	0.5	188.3	7.5	even	ok
823.512421	1	C50H72NaO8	100.00	823.511940	0.6	0.5	284.8	14.5	even	ok

yangbin_YK14_pos_12_01_3106.d
Bruker Compass DataAnalysis 4.1 printed: 6/12/2017 11:40:31 AM by: SCSIO Page 1 of 1

Figure S19: ^{1}H-NMR (700 MHz, CD_3OD) Spectrum of Compound **4**

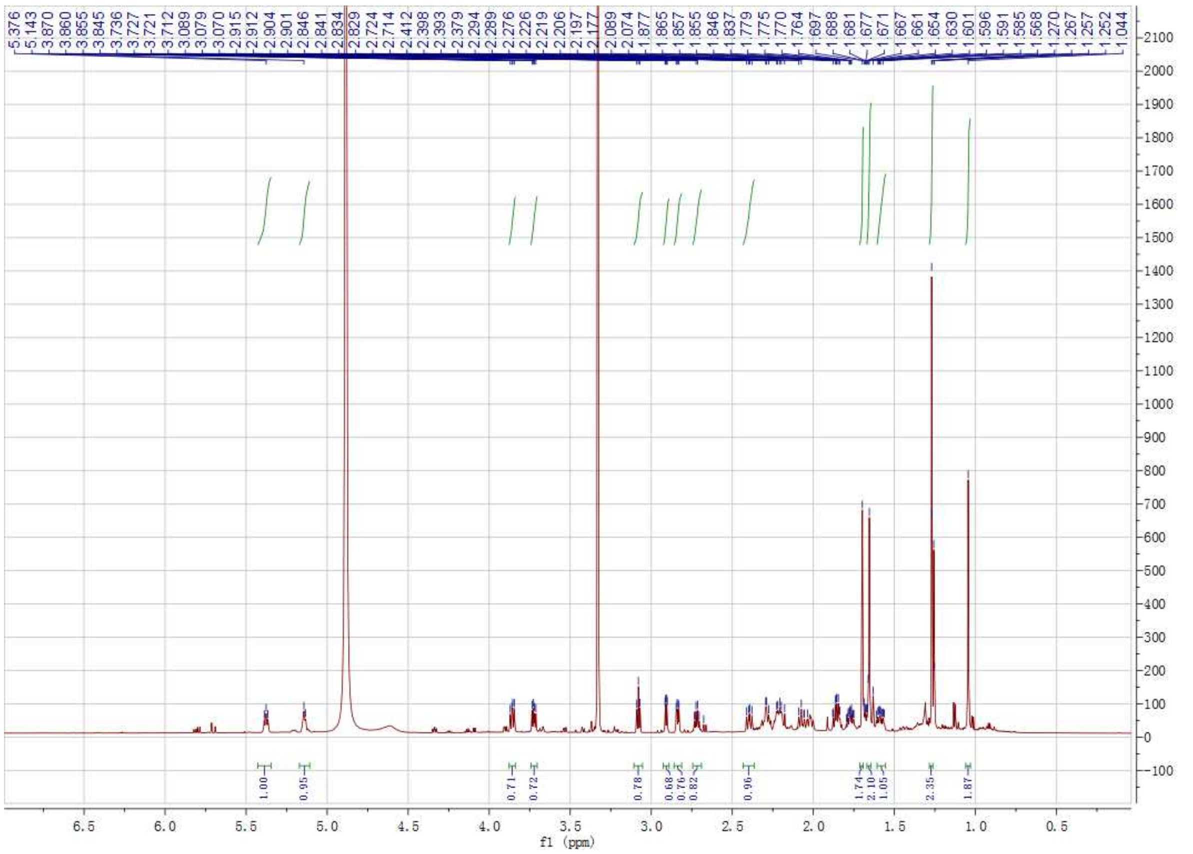

Figure S20: ^{13}C-NMR (175 MHz, CD_3OD) Spectrum of Compound **4**

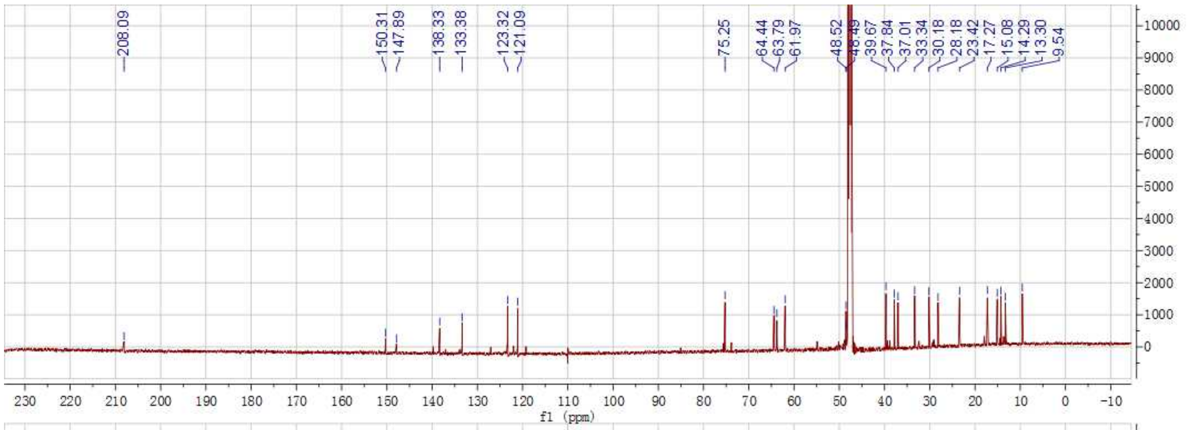

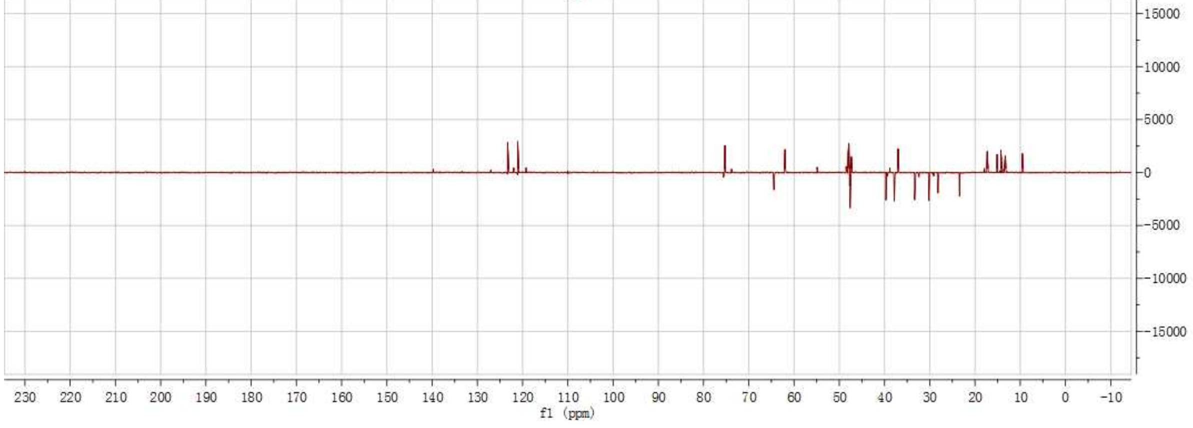

Figure S21: HMQC (700 MHz) Spectrum of Compound **4**

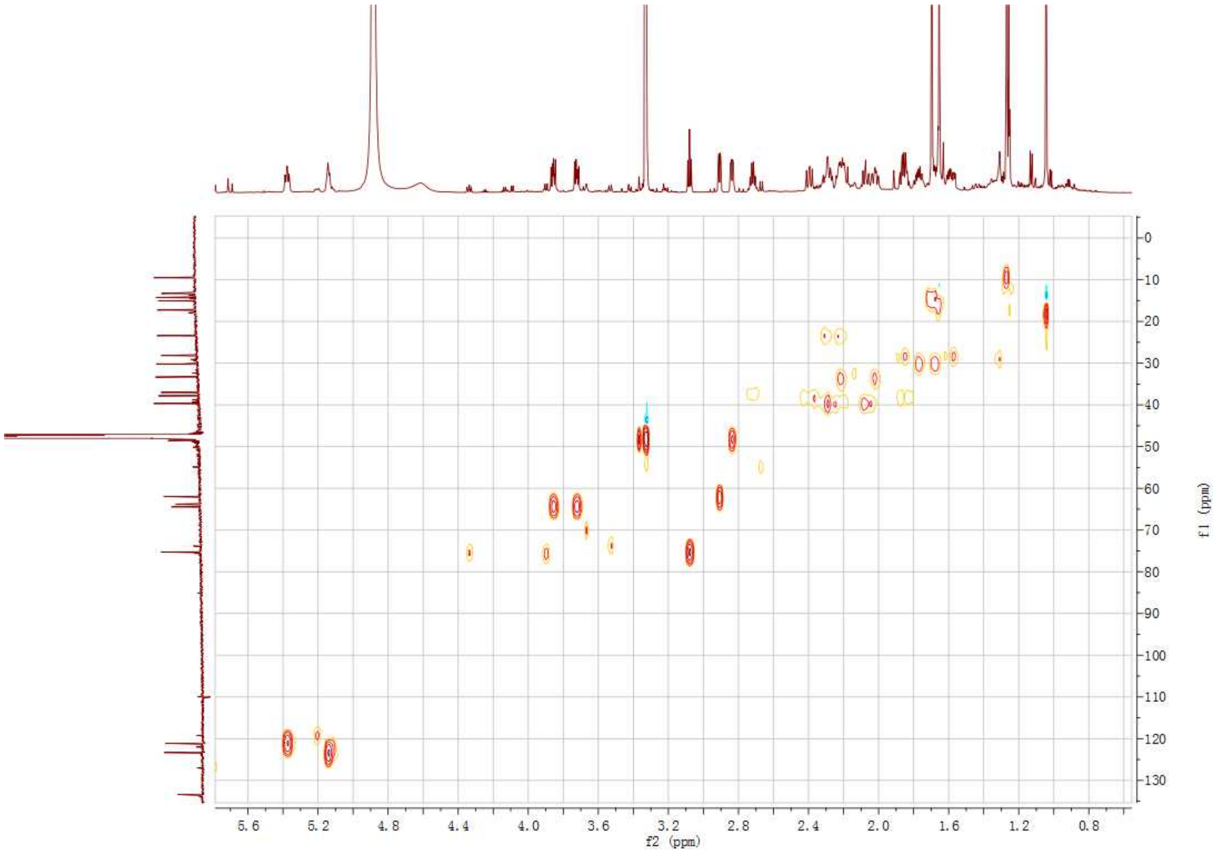

Figure S22: HMBC (700 MHz) Spectrum of Compound **4**

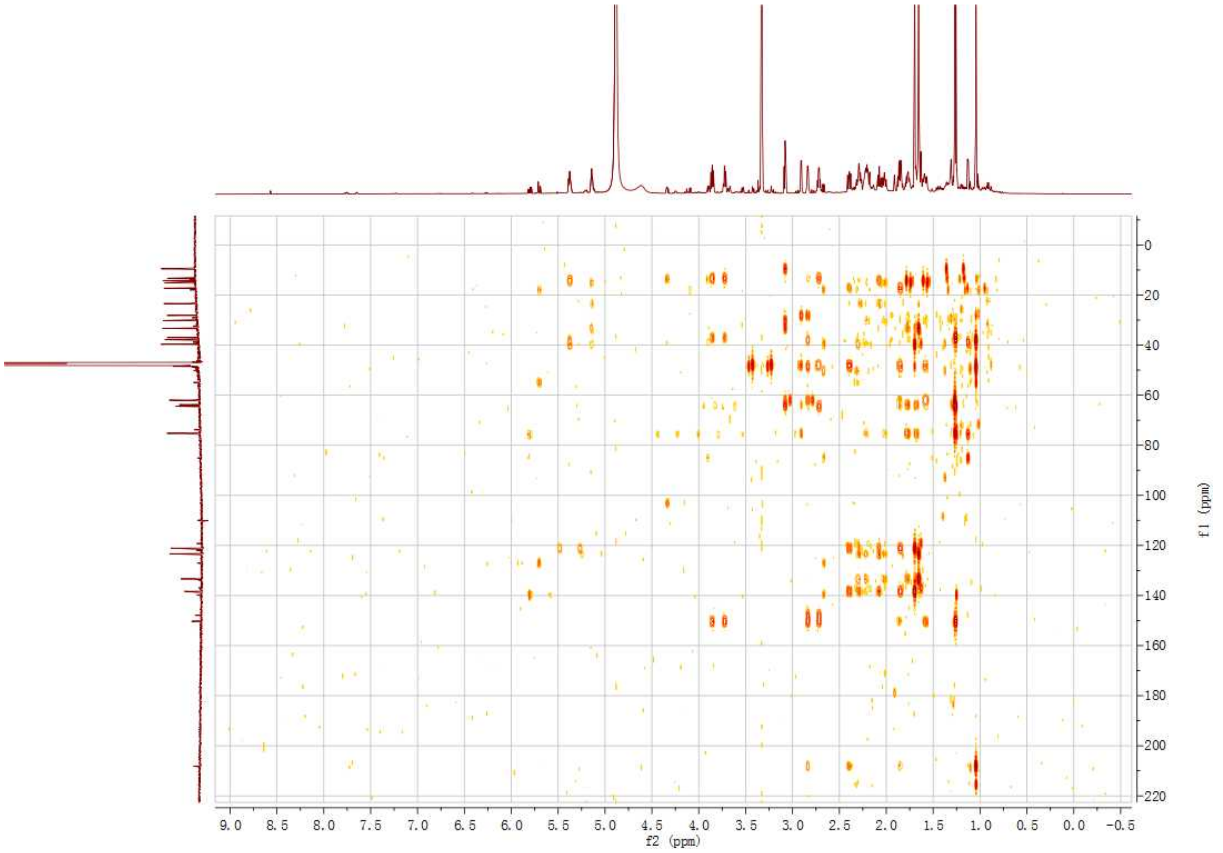

Figure S23: NOESY (700 MHz) Spectrum of Compound **4**

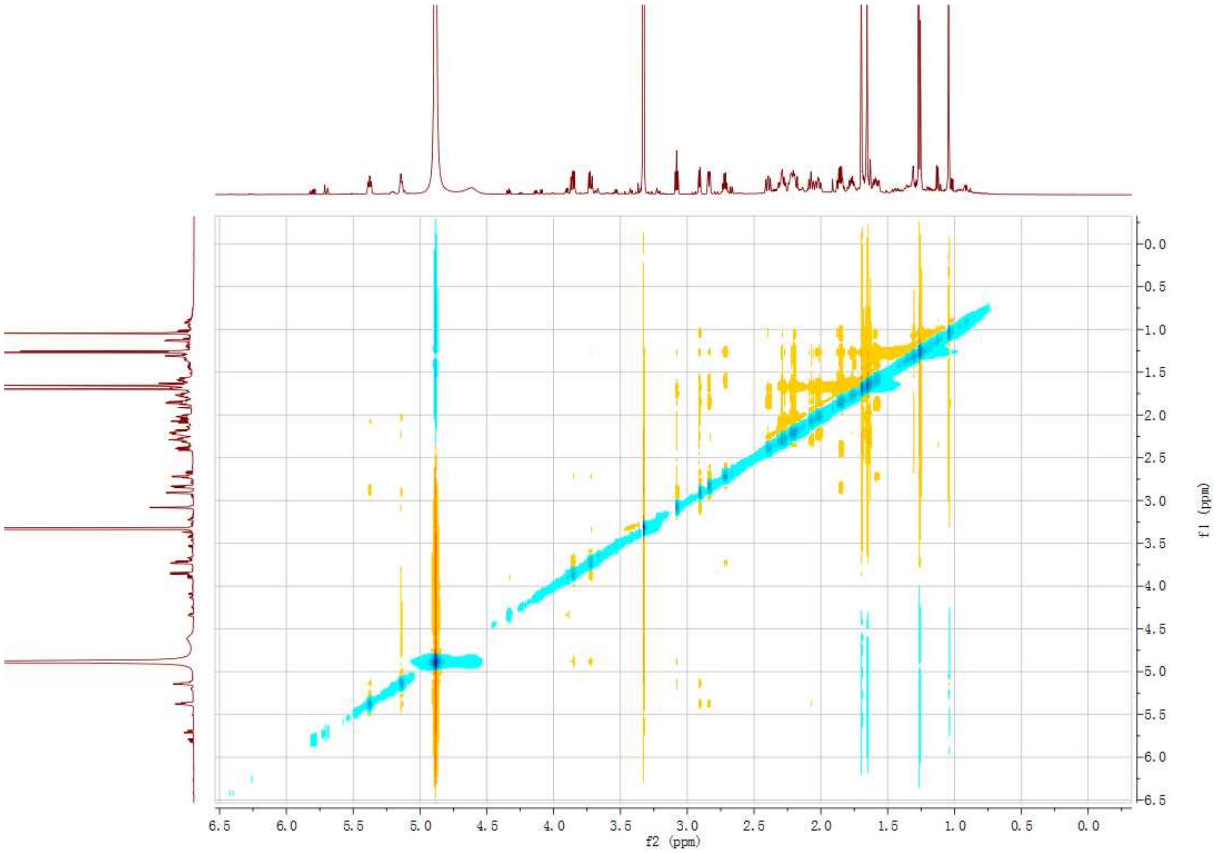

Figure S24: HRESI-MS Spectrum of Compound **4**

Mass Spectrum SmartFormula Report

**Analysis Info**		Acquisition Date	6/12/2017 11:23:26 AM
Analysis Name	D:\Data\MS\data\201706\yangbin_YK22_pos_15_01_3109.d		
Method	LC_Direct Infusion_pos_70-500mz.m	Operator	SCSIO
Sample Name	yangbin_YK22_pos	Instrument	maXis 255552.00029
Comment			

Acquisition Parameter

Source Type	ESI	Ion Polarity	Positive	Set Nebulizer	0.4 Bar
Focus	Active	Set Capillary	4500 V	Set Dry Heater	180 °C
Scan Begin	70 m/z	Set End Plate Offset	-500 V	Set Dry Gas	4.0 l/min
Scan End	1500 m/z	Set Charging Voltage	0 V	Set Divert Valve	Waste
		Set Corona	0 nA	Set APCI Heater	0 °C

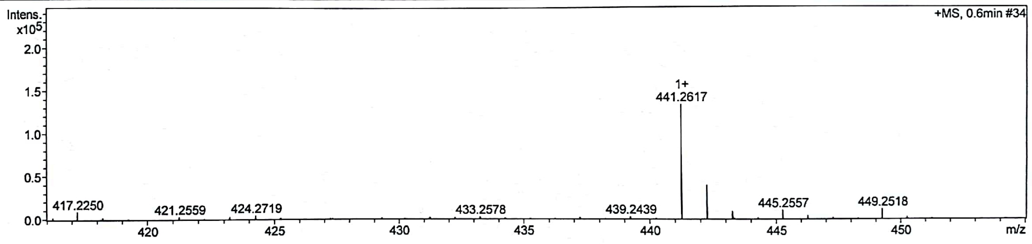

Meas. m/z	#	Ion Formula	Score	m/z	err [ppm]	err [mDa]	mSigma	rdb	e⁻ Conf	N-Rule
441.261749	1	C25H38NaO5	100.00	441.261145	-1.4	-0.6	14.4	6.5	even	ok
859.533221	1	C50H76NaO10	100.00	859.533069	0.2	0.2	67.0	12.5	even	ok

yangbin_YK22_pos_15_01_3109.d
Bruker Compass DataAnalysis 4.1 printed: 6/12/2017 11:47:54 AM by: SCSIO Page 1 of 1

Figure S25: Single-crystal X-ray structures of compound **6**

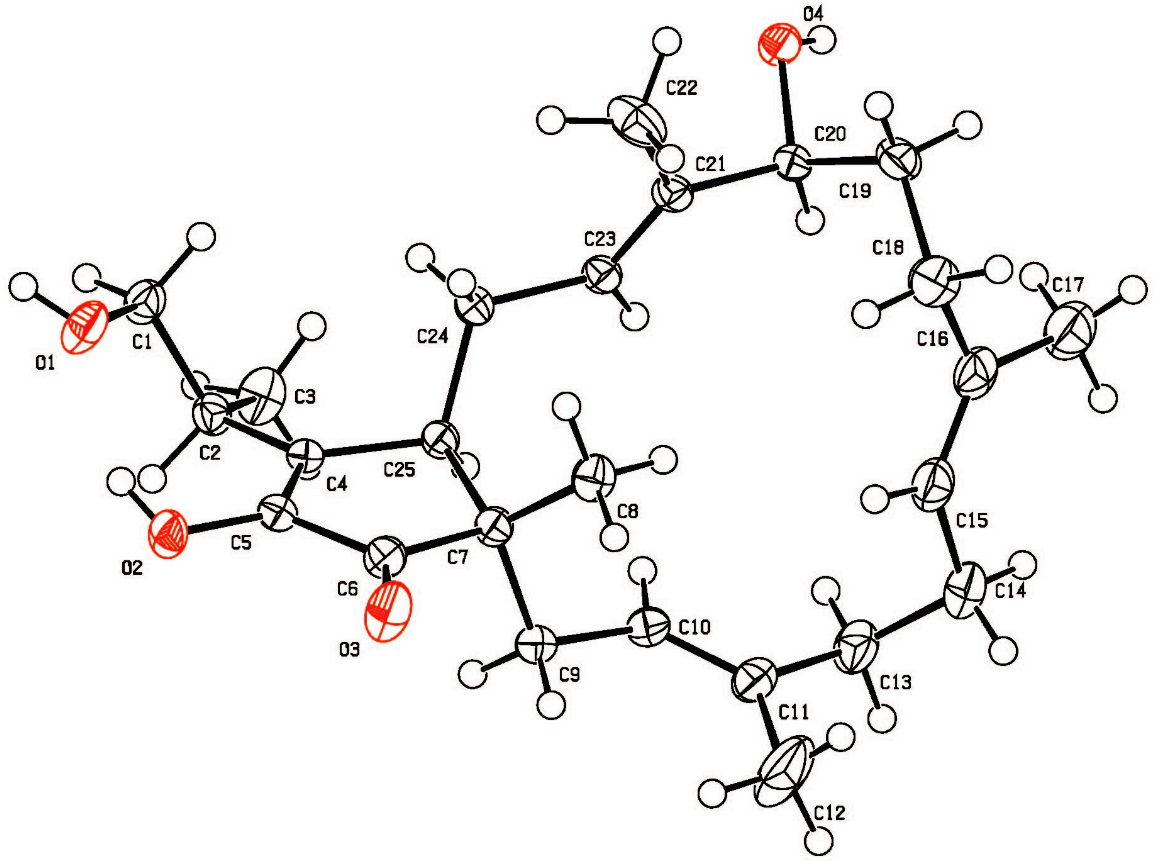

Figure S26: Effect of compounds **2**, **5**, and terpestacin (**6**) on the expression levels of HIF-1α and CD133 in U87MG-derived GSCs

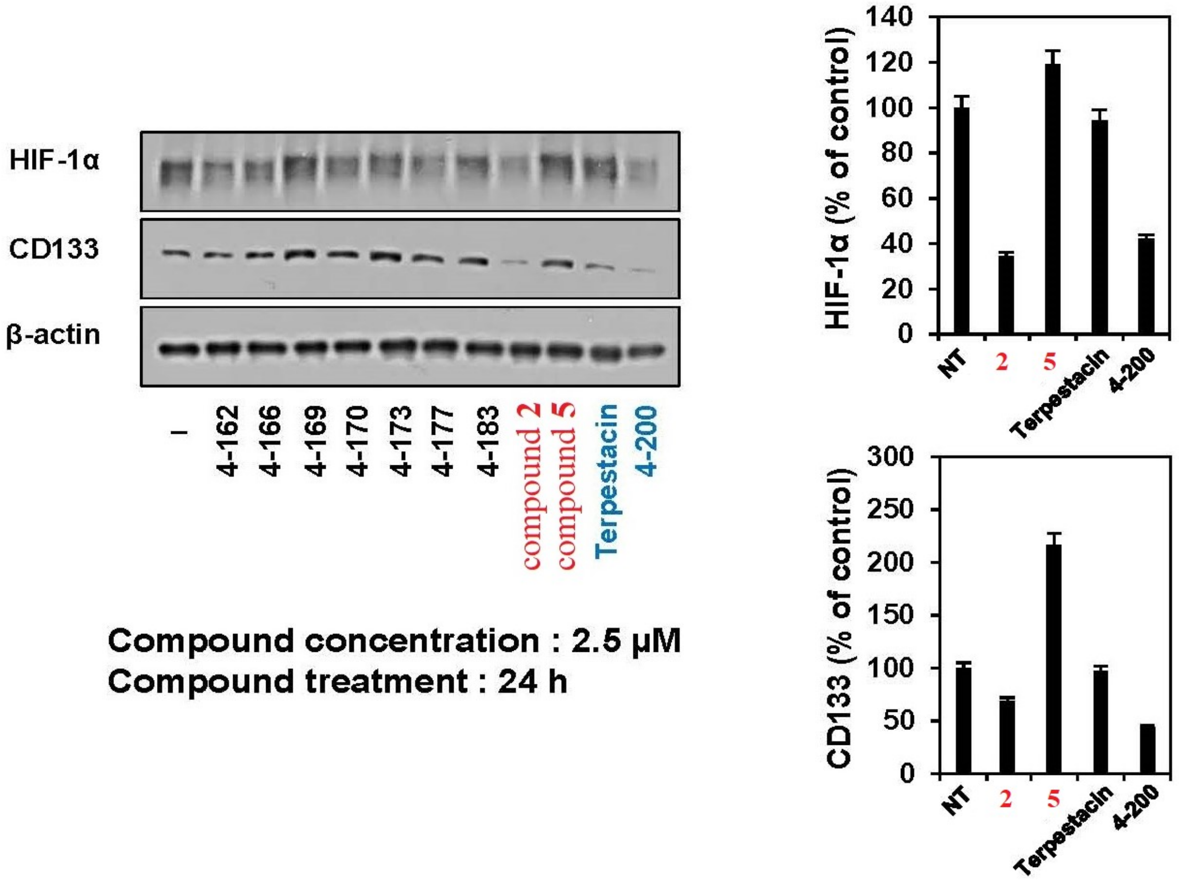

Supplement: Supplementary file 1 [file molecules-28-07246-s001.zip › molecules-2660196-supplementary.pdf]
